# Sodium selenite preserves rBM-MSCs’ stemness, differentiation potential, and immunophenotype and protects them against oxidative stress via activation of the Nrf2 signaling pathway

**DOI:** 10.1186/s12906-023-03952-7

**Published:** 2023-04-25

**Authors:** Bahareh Rahimi, Mohammad Panahi, Hajie Lotfi, Mostafa Khalili, Astireh Salehi, Neda Saraygord-Afshari, Effat Alizadeh

**Affiliations:** 1 Department of Medical Biotechnology, Faculty of Allied Medical Sciences, Iran University of Medical Sciences (IUMS), Shahid Hemmat Highway, Tehran, 1449614535 Iran; 2 Department of Medical Biotechnology, Faculty of Advanced Medical Sciences, Tabriz University of Medical Sciences, Tabriz, Iran; 3 Student Research Committee, Tabriz University of Medical Sciences, Tabriz, Iran; 4 Cellular and Molecular Research Center, Research Institute for Prevention of Non-Communicable Diseases, Qazvin University of Medical Sciences, Qazvin, Iran; 5 Biology Department, Islamic Azad University, Sanandaj Branch, Sanandaj, Iran

**Keywords:** Mesenchymal stem cells, Reactive oxygen species (ROS), Selenium selenite, Stemness, Nrf pathway

## Abstract

**Background:**

The physiological level of reactive oxygen species (ROS) is necessary for many cellular functions. However, during the *in-vitro* manipulations, cells face a high level of ROS, leading to reduced cell quality. Preventing this abnormal ROS level is a challenging task. Hence, here we evaluated the effect of sodium selenite supplementation on the antioxidant potential, stemness capacity, and differentiation of rat-derived Bone Marrow MSCs (rBM-MSCs) and planned to check our hypothesis on the molecular pathways and networks linked to sodium selenite’s antioxidant properties.

**Methods:**

MTT assay was used to assess the rBM-MSCs cells’ viability following sodium selenite supplementation (concentrations of: 0.001, 0.01, 0.1, 1, 10 µM). The expression level of OCT-4, NANOG, and SIRT1 was explored using qPCR. The adipocyte differentiation capacity of MSCs was checked after Sodium Selenite treatment. The DCFH-DA assay was used to determine intracellular ROS levels. Sodium selenite-related expression of HIF-1α, GPX, SOD, TrxR, *p*-AKT, Nrf2, and p38 markers was determined using western blot. Significant findings were investigated by the String tool to picture the probable molecular network.

**Results:**

Media supplemented with 0.1 µM sodium selenite helped to preserve rBM-MSCs multipotency and keep their surface markers presentation; this also reduced the ROS level and improved the rBM-MSCs’ antioxidant and stemness capacity. We observed enhanced viability and reduced senescence for rBM-MSCs. Moreover, sodium selenite helped in rBM-MSCs cytoprotection by regulating the expression of HIF-1 of AKT, Nrf2, SOD, GPX, and TrxR markers.

**Conclusions:**

We showed that sodium selenite could help protect MSCs during *in-vitro* manipulations, probably via the Nrf2 pathway.

**Supplementary Information:**

The online version contains supplementary material available at 10.1186/s12906-023-03952-7.

## Introduction

Mesenchymal stem cells are an ideal source for fundamental studies in stem cell biology and many regenerative medicine applications [1, 2]. MSCs are available in adulthood, can be extracted from different tissues with no ethical concerns, have high genetic stability, rapid proliferation, and have multipotent differentiation capacity [3-5]. However, some challenges exist regarding the quality of MSCs used in clinical approaches and cell-based therapies, which can affect the outcomes of the applications of MSCs in clinical and basic research [6]. Among these challenges, a critical issue is a high level of oxidative stress (OS) in the culturing conditions, which is associated with an increased rate of apoptosis, cell inactivation, phenotypic alterations, reduced stemness and differentiation capability, and aging. A disproportion between the production of reactive oxygen species (ROS) and the antioxidant defense system of cells is defined as OS [7, 8]. OS can predispose senescence by upregulation of protein peroxides and increasing the rate of DNA and lipids oxidation, which leads to a wide range of molecular and cellular modifications [9-11].

Therefore, culture conditions for MSCs must be controlled to adjust the normal ROS level and maintain the efficacy of MSCs [12-14]. One approach is to supplement the culture media of MSCs with antioxidants [15]. Antioxidant agents are classified into several categories: enzymatic/non-enzymatic, hydrophilic/hydrophobic, endogenous/exogenous, and so forth. Enzymatic antioxidants primarily serve as intracellular protection against OS. Their function is to eliminate oxidant molecules and convert ROS into harmless entities. Superoxide dismutase (SOD), thioredoxin reductase (TrxR), and glutathione peroxidase (GPX) are the three known enzymes of this category. Non-enzymatic antioxidants encompass a great collection of molecules such as vitamins (A, C, E, and K), cofactors (Q10), minerals (Zn, Se, and others), uric acid, bilirubin, and albumin. Generally, these non-enzyme antioxidants do their function by deactivating free radicals, ROS, and other oxidating molecules [16-18]. The cellular antioxidant defense system functions through three main steps: (i). prevention (by agents such as GPX), (ii). interception (e.g., by TrxR), and (iii). repair [19].

Selenium (Se) is a trace nonmetal element essential for human nutrition [20]. According to the NIH fact sheet for health professionals (https://ods.od.nih.gov/factsheets/Selenium-HealthProfessional/) the average daily selenium intake in Americans adults ranges from 134 to 93 mcg from foods for men and women respectively. These data raise to 151 t0 108 considering fool and supplements in nutrition regimen. Se is a fundamental component of seleno-proteins and some antioxidant enzymes, such as GPX and TrxR. Therefore, Selenium is an excellent component in the first and second levels of the antioxidant defense system [21-23]. Also, Selenium is an available and affordable agent with appropriate water solubility compared to other antioxidant agents. All these make Selenium an available and favorable choice for cell therapy and regenerative medicine applications [24].

Compared to other fields of medical and biological studies, the application of animal models in any type of stem cell-related research is inevitable. There are many debates and questions about the various aspects of stem cells’ applications, including choosing the best and most ethically acceptable cell type and answering many concerns about the high regenerative and developmental potential of stem cells that make them susceptible to hyperplasia and oncogenic transformation [25, 26]. In this context, consistency across different animal and human models and *in-vitro*, *ex-vivo*, and *in-vivo* experiments is another point that must be considered [27, 28]. In comparison to other animal model cells, rats are cheaper and their physiology, genetics, and cell processes are more close to humans and exhibited advantages in preclinical effectiveness studies resembling human stem cells [29]. Therefore, when they are used as model cells, the obtained data could be generalized to human cells.

Hence, in the following, we will mention some points regarding the application of Selenium to improve Bone Marrow MSCs (BM-MSCs) potency in animal and human models.

There are pieces of evidence indicating that selenium insufficiency is associated with diminished selenoproteins expression, increased inflammatory cytokines production, and oxidative stress in both animal and human models; on the other hand, selenium supplementation can help to decrease these inflammatory changes [30-32]. For example, thanks to the studies performed in animal models, at present, we know that due to its antioxidant properties, selenium alone or in combination with other antioxidants, such as vitamin E, can reduce OS-related damages in animal models of colitis [33, 34]. Moreover, we know that a low level of selenium is associated with several diseases in both animal and human models, such as cardiovascular diseases [35], type 2 diabetes [36], cancer [37], and neurological function [38]. All these observations have started an interest in selenium, and its derivatives, utilization as a defensive agent in various types of disorders, such as diabetes [39], nephropathy [40], and cancer [41]; also there are many kinds of research and trials on this issue [42, 43]. For example, in polycystic ovary syndrome (PCOS) rat models, sodium selenite has shown a significant effect on the restoration of GPX and has beneficial impacts on reproductive outcomes and oxidative stress markers [44-46].

However, as mentioned above, although several recent studies have described many beneficial effects of sodium selenite on MSCs derived from bone marrow [47, 48], adipose [49], and umbilical cord tissues [50], only sparse reports exist on the effects of sodium selenite on the stemness-associated marker genes, surface CD markers, and the selenium-linked OS signaling pathways. Hence, this study aimed to investigate the effects of Selenium supplementation on the viability of rBM-MSCs, stemness capacity (based on the expression level of *OCT-4*, *NANOG*, and *SIRT1* genes [51]), adipocyte differentiation potential, CD markers profile, intracellular ROS, and expression of some relevant proteins that were chosen by reviewing the bulk of relevant previously-published papers and considering the results of in-silico network analysis.

## Materials and methods

### Chemicals and reagents

The entire reagents and chemical materials utilized in this work were of analytical grade. Propidium iodide (PI), Ribonuclease A, 3 (4,5-dimethyl-2-thiazolyl)-2,5-diphenyl-2H-tetrazolium bromide (MTT), Dimethyl sulfoxide(DMSO) were purchased from Sigma–Aldrich (St. Louis, MO). Fetal bovine serum (FBS), Dulbecco’s Modified Eagle’s Medium (DMEM), Penicillin Streptomycin solution, and trypsin were purchased from Gibco Invitrogen (Grand Island, NY). L-Glutamine, β-glycerophosphate (10 mM) (Sigma), Alizarin Red S (Sigma), and dexamethasone (Sigma). Sodium selenite (Sigma), DMEM medium (Bioidea, Iran), Oil Red (Sigma-Aldrich), Trizol reagent (Thermo Scientific), Syber Green Master Mix (Thermo Scientific), CD105-Percp-Cy5.5 (Biolegend), CD44-PE (eBioscience), CD45-FITC (BD Bioscience), and CD31-PE (eBioscience). Euthanasia chamber (Orchid Scientific).

### Isolation, culture, and characterization of rBM-MSCs

All procedures related to rats in this research were approved by the Tabriz University of Medical Sciences Research Ethics Committee (No. IR.TBZMED.REC.1399.036). In this regard, we acted according to the well-accepted method of sacrificing and expression of some relevant proteins that were chosen sacrificing animal models guidelines of Tabriz University of Medical Sciences Animal models Ethics and related international guidelines (https://researchvice.tbzmed.ac.ir/?pageid=42). The rats (No = 3), purchased from Animal house, Faculty of Veternary, Tabriz University. They were in aged three to five weeks, and about 250 g, were used for the isolation of bone marrow-derived mesenchymal stem cells. A euthanasia procedure was performed in a way that the rat rapidly lost consciousness without any pain or sensing any suffering. The rats were located in an anesthesia CO2 chamber. Then, anesthetized for about 6 min. Subsequently, the rats were picked up from the chamber at the time they stopped their motor activity and their blinking rate ended. Next, the rats were laid down on the operation bench and killed by cervical dislocation of rats. The femur and tibia bones were cut off the soft tissues around them were detached [52]. rBM-MSCs were isolated from the rats’ bone marrow in line with the earlier published protocol with minor changes [53]. The femur’s epiphysis was cut off from both ends in a biosafety cabinet, and the bone marrow was inserted with a syringe needle into one end of the bone. Then flushed out with serum-free DMEM/F12 medium (10 mL) mixed with 100 μg/ml streptomycin and 200 U/ml penicillin into a 60 mm culture dish. The cell suspension was achieved by pulling the marrow into syringes three times in a series employing decreasing-sized needles. The rBM-derived cell suspension was centrifuged at 1000xg. These cells were then incubated at 37 °C in a complete medium under the standard condition of 95% humidity and 5% CO_2_ to reach 70% cell confluency. According to the routine cell culture protocol, media were refreshed twice a week.

Immunophenotyping by flow cytometry was used to characterize the rBM-MSCs surface CD profile. For this purpose, the extracted rBM-MSCs were subcultured in 6 well format plates in routine cultured and harvested at 70% area confluence. Approximately 2 × 10^5^ cells were transferred to FACS tubes and washed with phosphate-buffered saline (PBS). These cells were then incubated with solutions containing phycoerythrin (PE)- or fluorescein isothiocyanate (FITC)- conjugated antibodies against rBM-MSC markers. Four fluorescent dye-conjugated Flow cytometry-specific antibodies, including anti-CD34-PE, anti-CD45-FITC, anti-CD90-FITC, and anti-CD73-PE, were used for cell staining in the dark. Finally, the presence of the rBM-MSCs specific surface antigens was assessed by a flow cytometer (BD FACS Caliber; BD Biosciences, San Jose, CA, USA), and Cell Quest software was used to analyze the obtained data (BD Biosciences).

### Investigation of differentiation potential of rBM-MSCs

Adipogenesis and osteogenesis potential of extracted rBM-MSCs were investigated to confirm their multipotency. The confluent rBM-MSCs cells were harvested and counted, then a density of 5 × 10^4^ cells per well was seeded in a 6-well plate vessel in an expansion medium with a base of DMEM enriched with 10% of fresh fetal bovine serum (FBS), Penstrp (Gibco), 1X ITS (Sigma), and dexamethasone 1 × 10^–6^ M at standard Co2 incubator. When cells fill 80% of the wells’ area, the medium mentioned above was replaced with either osteogenic or adipogenic differentiation induction cocktail media.

To evaluate the rBM-MSCs potential for adipocyte differentiation, the cells were cultured for 14 days in the differentiation medium. Adipogenesis induction medium consisted of 10% FBS, 0.5 mM 3-isobutyl-1-methylxanthine (IBMX), 100 µM indomethacin, 10 µg/ml insulin, 1 µM dexamethasone. Considering the company’s recommended instructions. Differentiation Media were changed twice weekly, and special care was taken not to disturb the differentiation monolayer. By 14 days, cells were fixed and stained with Oil Red which stains neutral lipids. Meanwhile, to evaluate the differentiation potential of rBM-MSCs to osteocytes, the cells were cultured for 21 days in the differentiation medium, considering the company’s recommended instruction. Osteogenesis induction medium contained Alpha-Minimum Essential medium (α-MEM) enriched with 10 mM β-glycerol phosphate, 10% FBS, and 0.1 μM dexamethasone (Bioidea, Iran) Louis, MO, USA) and 10% FBS [54].

After 21 days, the differentiated cells were subjected to Alizarin/Red staining solution (Bioidea, Iran) to detect the mineralization within the calcium-containing osteocytes. The final images of both experiments were then observed and acquired by inverted microscopy (Hund, Wetzlar, Germany).

In previous works, the effect of Se on osteogenesis and chondrogenesis was checked [55] therefore in the present work, we checked the effect of sodium selenite on adipogenesis. In this way, rBM-MSCs (P5) were seeded in 6 well plates and treated with 0.1 µM sodium selenite supplemented in a routine medium for 72 h, then the medium was replaced with an adipogenesis induction cocktail. The oil droplets after adipogenesis was quantified using our previously deep learning-based method [56]. The microscopic images of oil droplets produced after adipogenic differentiation in control or sodium selenite treated cells were quantified. The captured microscopic photos were divided into square drawings, and the count of lipid droplets were studied, based on the deep learning methodology [56].

### Evaluation of the optimal sodium selenite dosage and treatment course MTT assay and cell morphology observation

The tetrazolium-based MTT assay was employed to study the impact of different sodium selenite concentrations on the rBM-MSCs viability and proliferation. For this purpose, ~ 5000 rBM-MSCs were transferred to each well of a 96-well plate and incubated overnight. Cells were allowed to attach to the bottoms of microtiter wells and expand themselves in the expansion medium mentioned above. Then the medium was replaced with a series of low glucose DMEM media containing five different concentrations of sodium selenite (0.001, 0.01, 0.1, 1, and 10 µM) and further incubated for 24, 72 h. According to the previous report, the doubling time of the rBMSCs population is 49.9 ± 4.2 h [57] Therefore, in the present study, we performed a preliminary study by duration times less (24 h) and more than doubling time (72 h) and a series of concentrations. Following treatments, the media was aspirated from each well and discarded. 20 μl MTT solution in the fresh Selenium-free medium was added to each well and incubated in the dark for 4 h. After removing the supernatants, 200 μl of dimethyl sulfoxide (DMSO) (Sigma-Aldrich) was added to each well and incubated for about 20 min to completely dissolve the produced formazan crystals. Subsequently, the absorbance of each well was measured at the wavelength of 570 nm in an ELISA plate reader. Moreover, morphological analysis of cells before and after treatment with sodium selenite was examined by inverted microscopy (OLYMPUS IX71).

### Evaluation of stemness-related genes expression

The effect of sodium selenite treatment on the expression level of the three known stemness-related genes (i.e., *OCT*-4, *NANOG*, and *SIRT1*) was evaluated by qRT-PCR. For this purpose, the rBM-MSCs were cultured in a complete DMEM low glucose media supplemented with sodium selenite to the concentration of 0.1 µM. The control replicates were also included in which the rBM-MSCs were cultured in the same condition but without sodium selenite supplementation. After 72 h, cells from the treat and control groups were collected and used for total RNA extraction with Trizol reagent according to the manufacturer’s protocol. The RNA obtained from the Trizol extraction step was mixed with RNA-Free DNase to eliminate any DNA impurity. The concentration of the total extracted RNA for each replicate was then determined by the UV–Vis spectroscopy approach using NanoDrop (Thermo Scientific, Waltham, MA). The cDNA was synthesized using the Revert Aid cDNA synthesis kit from the Thermo Scientific company. The expression level of the three mentioned stemness-related genes was evaluated using cyber Green Master Mix and unique primers (Table 1) on a Rotor gene 6000 system (Corbett, Qiagen, Australia) at 40 cycles as follows: 95° C for 10 min, denaturation at 95° C for 10 min, annealing 60° C for the 30 s and extension step at 72° C for 30 s. The formula of fold chamg = 2^–∆∆Ct^ was applied to analyze the acquired CTs, and the rat glyceraldehyde-3-phosphate dehydrogenase (GAPDH) gene was referenced as the housekeeping gene. The control group expression (fold change) was normalized to 1 and the relative expression > 1 was considered as up-regulation and the 0 < relative expression < 1 as down-regulation for each gene.

**Table 1:** Primer sequences that were used for stemness gene expression in qPCR

Gene	Forward primer	Reverse primer
NANOG	GAGACTGCCTCTCCTCCGCCTT	GTGCACACAACTGGGCCTGA
OCT-4	CTGGAGAGGGATGTGGTTCG	AAGGGACCGAGTAGAGTGTG
SIRT1	CGCCTTATCCTCTAGTTCCTGTG	CGGTCTGTCAGCATCATCTTCC
GAPDH	TCAAGAAGGTGGTGAAGCAG	AGGTGGAAGAATGGGAGTTG

### Measurement of intracellular ROS level in live cells

The 2′,7′-dichloro-dihydro-fluorescein diacetate (DCFH-DA) assay was used to measure the level of intracellular ROS accumulation after 72 h incubation in a cell culture medium with the concentration of 0.1 µM sodium selenite and also the control group.

The rBM-MSCs were first seeded in a 6-well plate and treated for 45 min with 30 µM DCFH-DA in the dark. The DCFH-DA treated rBM-MSCs were collected by trypsinization and washed twice in PBS. The DCF fluorescence was then measured using a FACS cytometer by FACS analyses (BD FACS Calibur (BD Biosciences, San Jose, CA, USA)) at an excitation wavelength of 488 nm and DCF band-passed emission filter of 530 nm. The histograms of flow cytometry analysis were obtained using the FlowJo software (Cell QuestTM software, BD Biosciences). Then PI dye was added to the tubes containing the rBM-MSCs, to the final concentration of 1 μg/ml. The whole PI staining process was performed on ice and in the dark, and the tubes immediately proceeded to the cytometry analysis. PI fluorescent data were collected in the FL-3 channel (620 nm) of FACS (BD FACS Calibur (BD Biosciences, San Jose, CA, USA)).

### FACS characterization of the rBM-MSCs: effect of sodium selenite treatment

The rBM-MSCs treated with 0.1 µM of sodium selenite and controlled cells were characterized by FACSCalibur (Becton Dickinson). Antibodies and isotypes were used as follows: IgG1-FITC -Isotype control, IgG1-PE -Isotype control, and IgG1-PerCP. Cy5.5-Isotype control, CD105- (Biolegend), CD44-PE (eBioscience), CD45-FITC (BD Bioscience), and CD31-PE (eBioscience).

### Western blot analysis

Western blot analysis was performed for AKT, P38, SIRT1, SOD, GPX, TrxR, HIF1-α, and Nrf2, which are among the key proteins involved in the regulation of oxidative stress. The analysis was carried out as previously described with some modifications. Briefly, the cell lysates were mixed with an equal volume of the 5X Laemmli sample buffer. The final protein concentrations were determined by the Bradford assay at OD_595_. The protein contents of the fifth passage of the rBM-MSCs (15 μg per lane) in the Laemmli sample buffer were boiled for 5 min to ensure complete solvation and then separated using an SDS-PAGE approach. The final SDS-PAGE pattern of these proteins was then electrotransferred onto a 0.2 μm immune-Blot™ polyvinylidene difluoride (PVDF) membrane (Cat No: 162–017,777; Bio-Rad Laboratories, CA, USA) in a transfer apparatus. At the next step, PVDF membranes were subjected to blocking in a 5% BSA solution for 120 min (Cat No: A-7888; Sigma Aldrich, MO, USA) in 0.1% Tween 20 for 1 h. Then, the membranes were incubated in the anti-HIF-1α (Cat No: ab51608, Abcam), anti-GPX (Cat No: ab96257, Abcam), anti-AKT (Cat No: 680302, Biolegend), anti-Nrf2 (Cat No: ab62352, Abcam), anti-p38 antibody (Cat No: ab170099, Abcam), anti-SOD (Cat No: ab83108, Abcam), anti-TrxR (Cat No: sc-28321, Santa Cruz) and anti-beta actin-loading control antibodies (Cat No: ab8227; Abcam) for 1 h at room temperature. After the first incubation in the solutions of the primary antibodies, for washing of the membranes TBST solution was rinsed three times and subsequently exposed to goat anti-rabbit IgG H&L HRP-conjugated (Cat No: ab6721; Abcam) and goat anti-mouse IgG (HRP) (Cat No: ab97240; Abcam) secondary antibodies. Afterward, the membranes were incubated with enhanced chemiluminescence (ECL)western blot reagents for 1–2 min and visualized using an enhanced chemiluminescence system. The expression of proteins was normalized to β-actin. The blots were then subjected to a densitometry analysis, and the relative concentration of the protein bands was measured using gel analyzer Version 2010 software (NIH, USA). To calculate the relative concentrations of each band, (Percentage area under the curve band/ the percentage area under the curve of actin band, based on the calculated values, the groups were compared as described previously [58].

#### Molecular function analysis by DAVID database

After analyzing our proteins with western blot, Entrez gene ID of AKT, P38, SIRT1, SOD, GPX, TrxR, HIF1-α, and Nrf2 were uploaded into the DAVID database (DAVID 6.8; http://david.abcc.ncifcrf.gov/) to identify their molecular functions.

### In-silico investigation of ROS stress

The protein–protein interaction (PPI) network analysis was performed using STRING (https://string-db.org/), to seek the interactions among AKT, P38, SIRT1, SOD, GPX, TrxR, HIF1-α, and Nrf2 in homo sapiens (Fig. 8B).

### Statistical analysis

The experiments were designed in at least three replicates for all tests. The obtained data were analyzed using SPSS software (version 16), and the results were stated as mean ± SD. Differences between the means of each group were evaluated using Student’s t-test or ANOVA. A confident *p*-value range (*p*-Value < 0.05) was accepted as statistically significant.

## Results

### rBM-MSCs isolation and validation

**Fig. 1:**
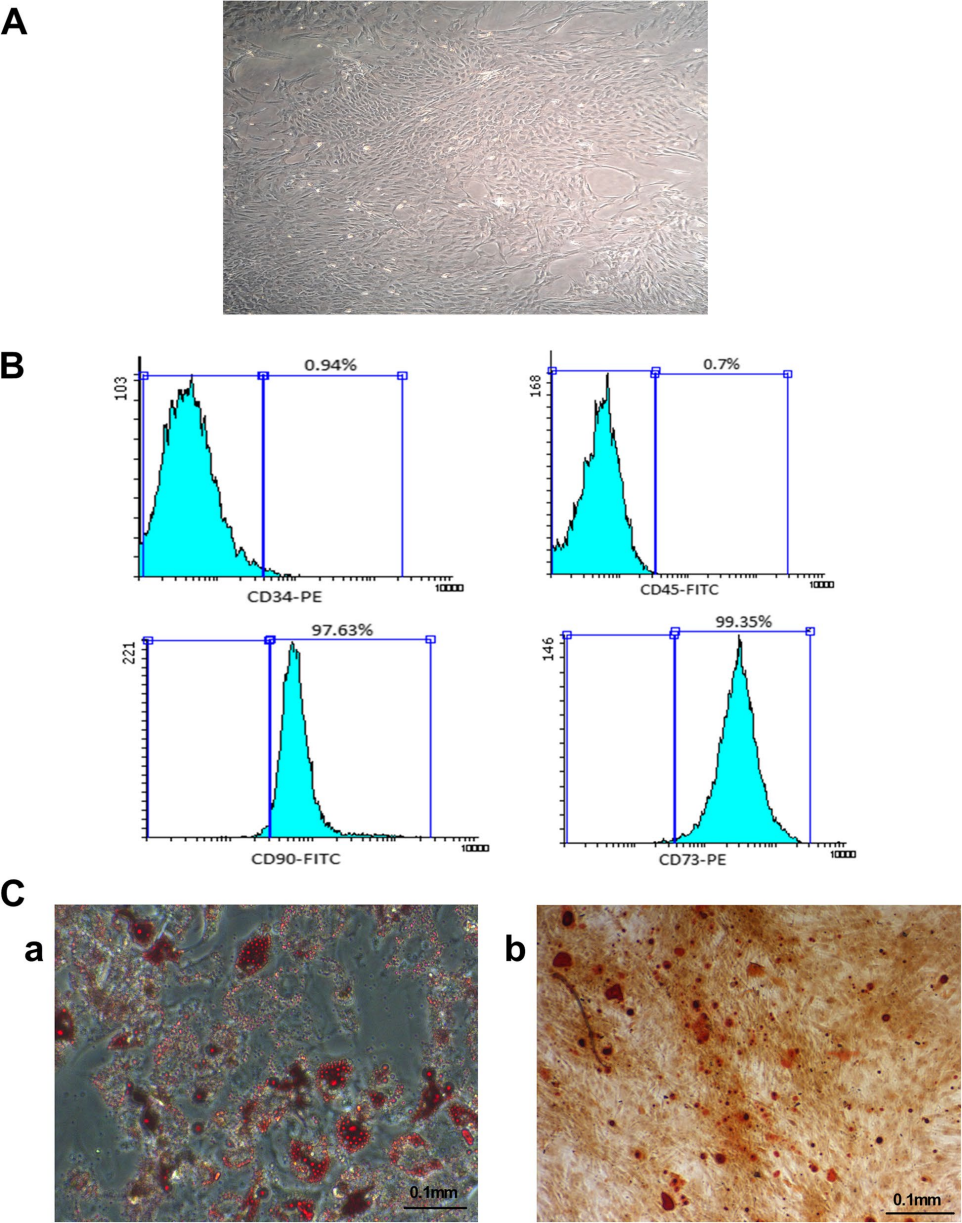
Isolation, cultivation and characterization of rBM-MSC. **A** The rBM-MSCs morphology was spindle shape, and they established adherent culture within one week. **B** Most (97.63%) of rBM-MSCs showed positive surface expression for CD90 as a surface marker in MSCs. Also, 99.35% of them were positively stained for CD73. An ignorable percentage (less than 1%) of the rBM-MSCs showed expression of CD34 and CD45, which are markers of hematopoietic lineages. **Ca** Confirmation of adipogenesis potential was done by the presence of detection of intracellular lipid droplets. **Cb** Confirmation of osteogenesis potential was done by the presence of mineralization particles stained red. All experiments were done in triplicate

In the first step, the morphology of the isolated cells was studied under an inverted light microscope which was spindle-shaped after 48 h in vitro culture and reached 60% confluency after a week (Fig. 1A). Based on the FACS analysis, the isolated rBM-MSCs’ surface marker profile was in accordance with standard CD markers of MSCs [59]. In particular, the isolated rBM-MSCs were positively stained for CD90 (96.21%) and CD73 (97.09%), but almost the majority of them didn’t express CD45 or CD34 (CD45 (0.7%) and CD34 (3.67%)) (Fig. 1B).

The rBM-MSCs were subjected to adipocyte differentiation to evaluate their multipotency capability. After 10 days of induction, lipid droplets were observed under a microscope, indicating the maintained adipogenesis potential of these cells. Subsequently, oil red staining was also performed and confirmed adipocyte differentiation (Fig. 1Ca). The osteoblast differentiation potential of rBM-MSCs was also assessed by exhibiting calcium deposits after the differentiation process. Accordingly, standard Alizarin red staining exhibited calcium accumulation detectable as red mineralization showing spots (Fig. 1Cb).

### Cell proliferation study findings

**Fig. 2:**
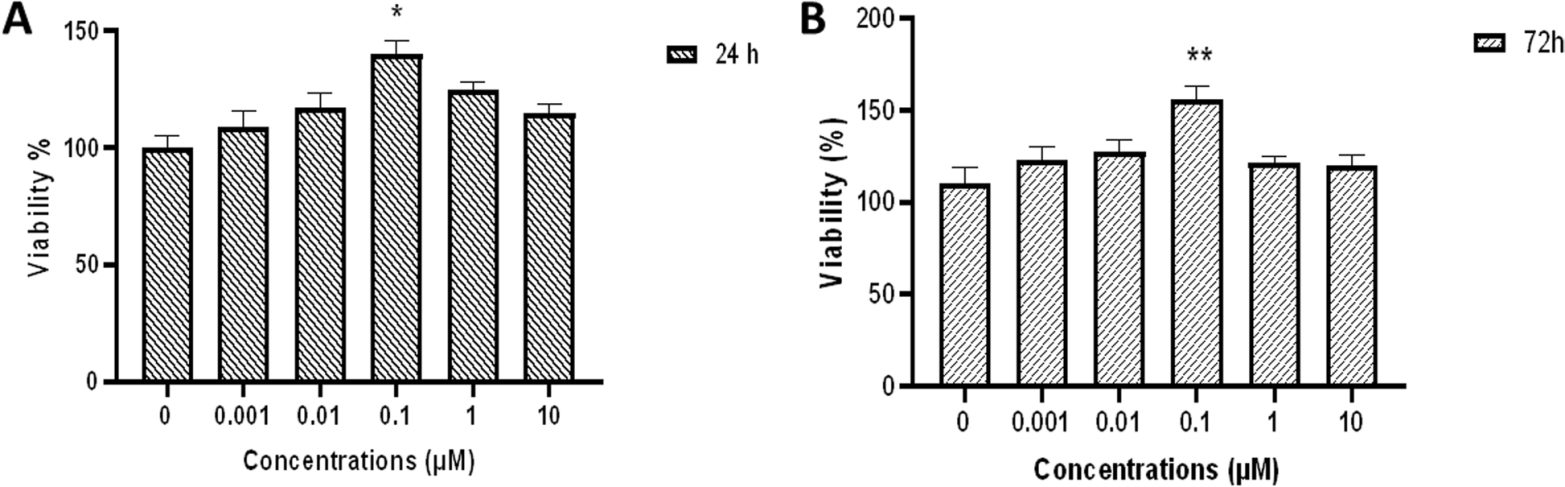
Effects of Sodium selenite on the viability of rBM-MSCs, determined by MTT assay after 24 (**A**) and 72 (**B**), hours incubation with different concentrations (0.001, 0.01, 0.1, 1, 10 µM) concentration in comparison to the control group. In the rBM-MSCs optimum OD was obtained at 0.1 µM of Sodium selenite, which showed the best viability and proliferation.*p < 0.05. Number of samples = 3, Data: Mean ± SD

Sodium selenite supplementation was performed in a range of 0.001 to 10 µM concentrations in the expansion medium of rBM-MSCs, and their proliferation was compared with the non-treated control group. The colorimetric proliferation MTT assay results demonstrated that the best concentration of sodium selenite for supporting the proliferation of the rBM-MSCs is 0.1 µM. Since the number of the cells increased significantly at concentrations up to 0.1 µM, while the impact of higher doses (0.001, 0.01, 0.1, 1, 10 µM) on proliferation potential was less when compared with 0.1 µM at different treatment intervals (24 h, 72 h) (Fig. 2). For the next experiments, 0.1 µM was used and other studies also reported the same dosage range: Yan et al. reported that 0.1 μM and 1 μM of Sodium selenite were able to promote the BMSCs proliferation and apoptosis, respectively. In addition, they found that using 0.1 μM of Sodium selenite significantly induce osteogenic and adipogenic differentiation via up-regulating associated factors such as LPL and PPRAG ( lipid factors) and RUNX2, COL1, and BGP ( as osteogenic factors) [47]. Ebert et al. showed that supplementation of selenite (0.1 μM) is an important countermeasure during oxidative stress in routine cell culture process due to increasing the anti-oxidative status of BMSCs by restoring basal GPx and TrxR, promoting mRNA expression of basal and ROS-stimulated SOD1, and finally decreasing the accumulation of ROS [48]. Che et al. concluded that in rats with Hashimoto’s thyroiditis (HT), the combination of Sodium selenite (0.5–20 µM) with adipose-derived mesenchymal stem cells (AMSCs) is able to accelerate the growth of AMSCs and their anti-oxidative potential by raising the glutathione level significantly. Moreover, Sodium selenite increased the level of inflammatory cytokines such as HGF (hepatocyte growth factor), TGF-β (transforming growth factor beta), and SCF (stem cell factor) [49].

### Gene expression

**Fig. 3:**
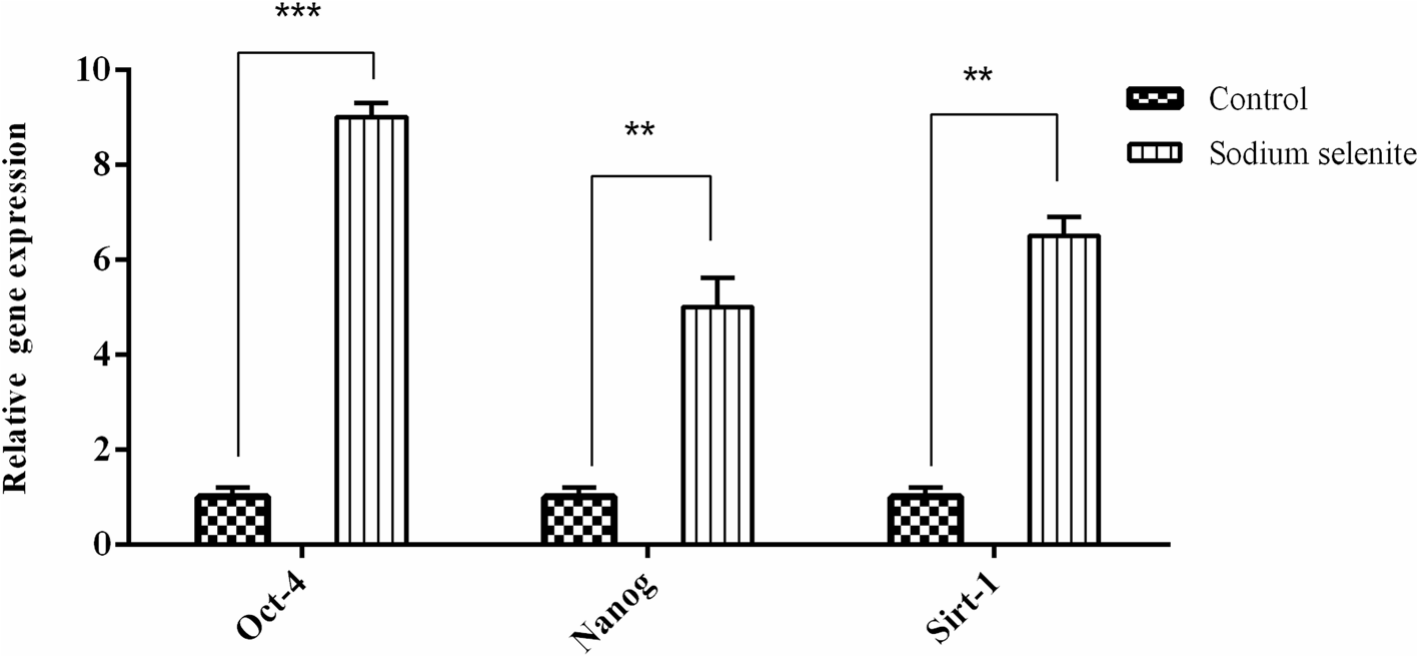
Relative gene expression of Oct-4, NANOG, and SIRT1 at 0.1 µM Sodium Selenite and control groups. The Oct-4, NANOG, and SIRT1 were significantly upregulated. The error bars show mean ± SD.***(*p*-Value ≤ 0.001), **(*p*-Value ≤ 0.01). Number of samples = 3, Data: Mean ± SD

The qPCR results showed that the concentration of 0.1 µM sodium selenite significantly improved the expression of OCT-4, NANOG, and SIRT1 in the treated rBM-MSCs compared to the untreated control group (*p*-Value < 0.01) (Fig. 3).

### Effect of Sodium Selenite on intracellular reactive oxygen species and cell viability

**Fig. 4:**
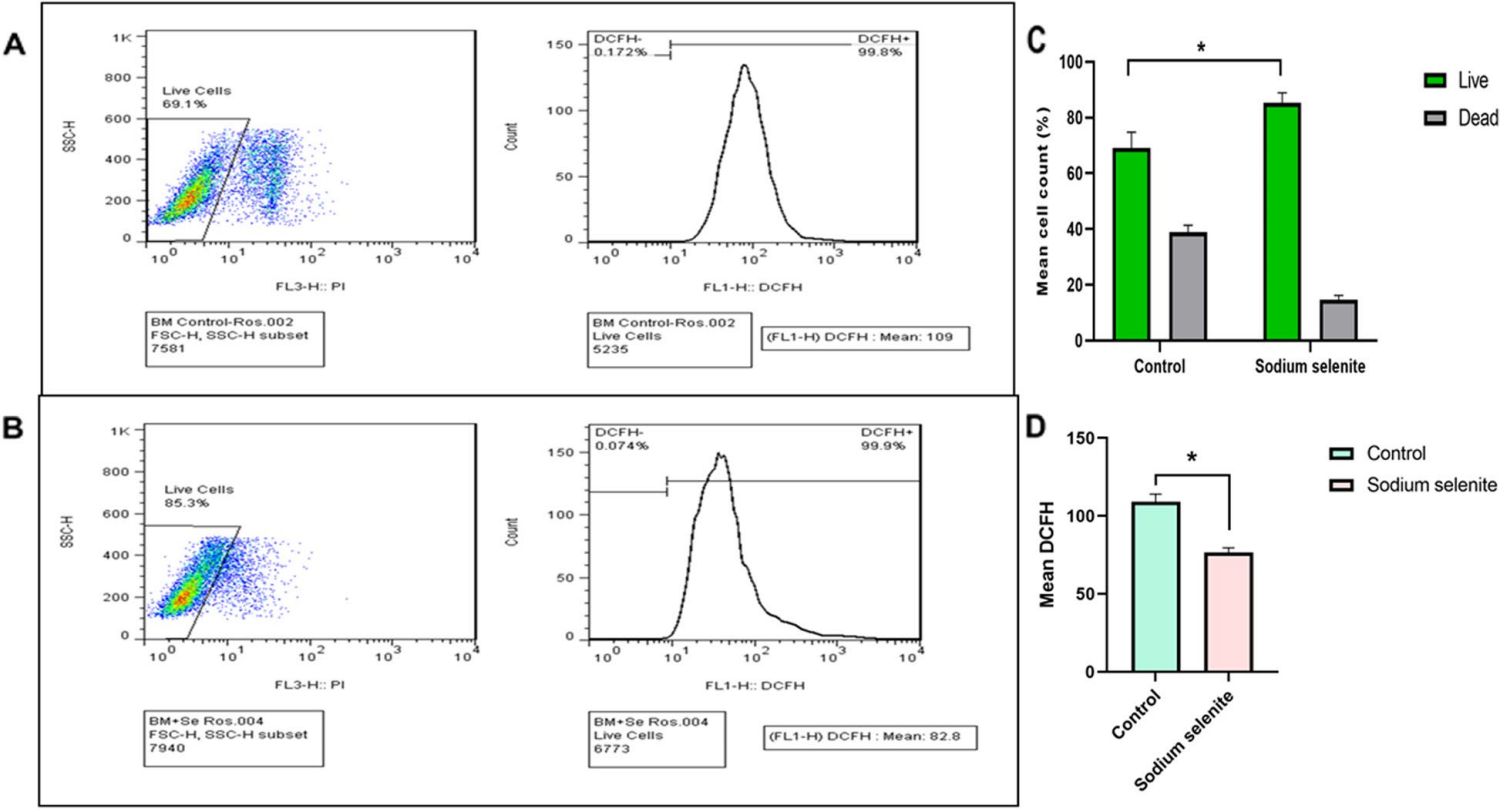
ROS generation and viability assessment by flow cytometry analysis. ROS generation in rBM-MSCs (**A**) cultured with Sodium Selenite (0.1 µM) (**B**). The ROS levels were determined by DCFH staining and flow cytometry. The viability of cells before and after Sodium selenite treatment was studied (**C**), which showed higher live cell populations after treatment. The ROS levels reduced after sodium selenite treatment (**D**). Number of samples = 3, Data: Mean ± SD

In the presence of Sodium selenite as an antioxidant, the level of the oxidized form of DCFH was decreased in the rBM-MSC, and treatment with sodium selenite (0.1 µM) can decrease the level of cellular oxidative stress below the value of untreated cells (*p*-value:0.05) (Fig. 4A-D). Also, along with the ROS detection method, the living cell population was evaluated by PI staining, and although it cannot penetrate the cell membranes of living cells, it can penetrate those of dying or dead cells. Before Sodium selenite supplementation, the counted live cells were -5235 ± 261, but after treatment, they counted 6773 ± 135.46 (Fig. 4C). The results showed that the percentage of live cells was 69.1% ± 5.7 in the untreated group, but it was increased to 85.3% ± 3.6 in the Sodium selenite-treated group, which shows the viability enhanced by Sodium selenite treatment (Fig. 4C).

### Morphological and FACS characterization of sodium selenite treated cells

Although in various studies for cell therapy applications using passage 3–5 of MSCs is recommended. A number of reports have proved that passage 5 of MSCs is at the border of initiating senescnce and reduction of stemness characteristics therefore could be examined as model cells to study the effects of antioxidants on stemness properties. Yang and colleagues documented that MSCs maintain their morphology and plasticity up to P5 but at later subcultures show irregular morphologies and sizes [51]. Even at lower passage number for instance in passage 3 MSCs showed reduced telomerase activity and chromosome anomalies may originating from in vitro expansion, which could impair stemness of them [60]. Also, study by Ridzuan et al., documented that the enzyme beta galctosidase as a senescence marker detected to become up regulated at passage 5. Besides G0/G1 arrested cell cycle because of cellular senescence at in vitro expansions were observed in passage 5 [61].

**Fig. 5:**
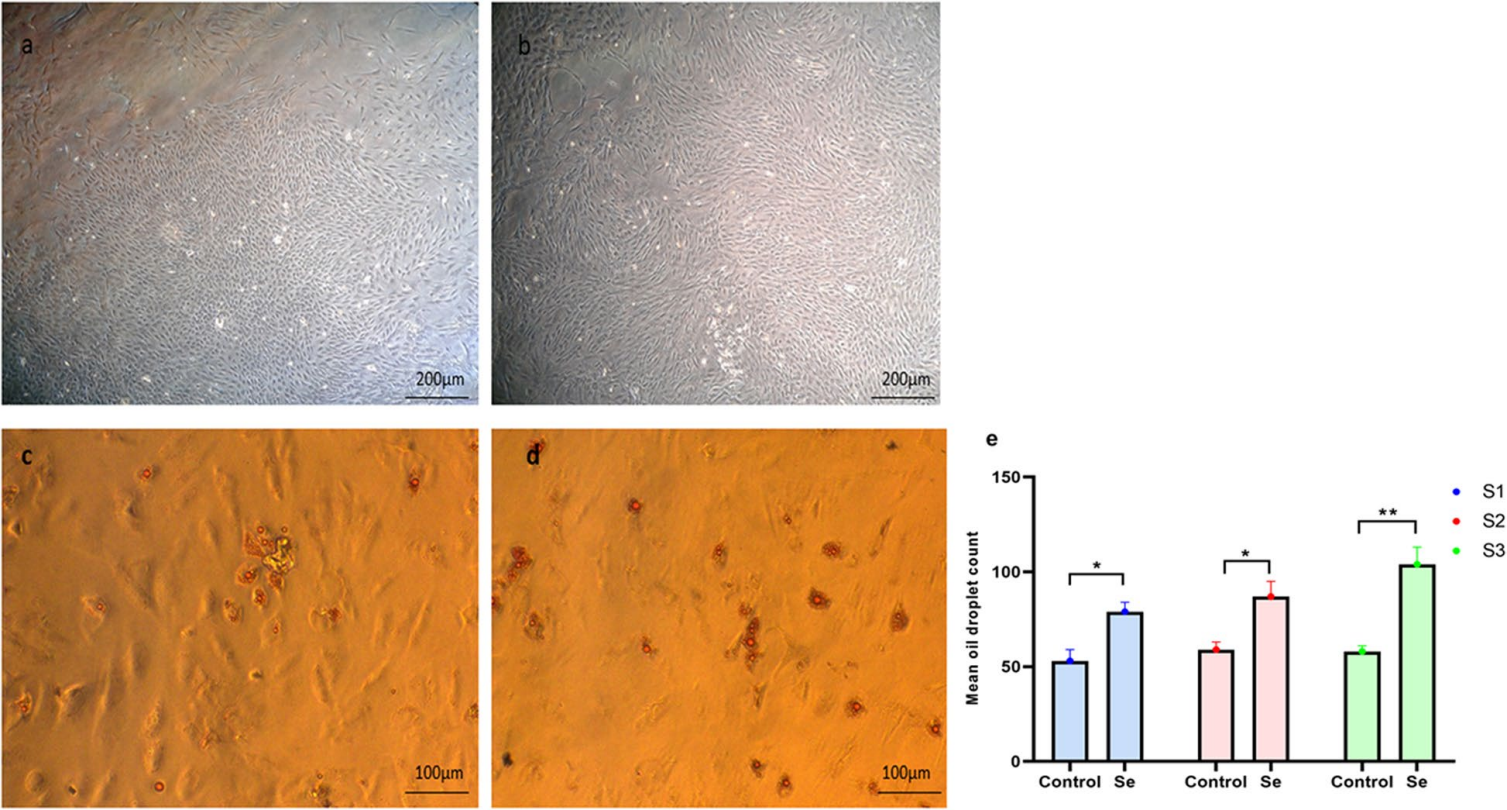
Morphological observation and adipogenic differentiation of control compared to Sodium selenite treated rBM-MSCs. Control rBM-MSCs (**a**) rBM-MSCs treated with sodium selenite exhibited spindle-shaped and elongated without significant morphological changes (**b**). rBM-MSCs subjected to adipogenesis (**c**), rBM-MSCs treated with sodium selenite showed increased lipid droplets after oil red o staining (**d**). The percentage of oil droplets were quantified and reported (**e**). Number of samples = 3, Data: Mean ± SD

P5 passages of rBM-MSCs were cultured with 0.1 µM of sodium selenite for 72 h, and in comparison, with untreated cells, no significant morphological changes were observable. Both groups had spindle-shaped and elongated cells Fig. 5 a, b. A comparison of the adipogenesis potential of sodium selenite pre-treated cells and those of controls (Fig. 5c and d, respectively) exhibited a higher percentage of oil droplets in sodium selenite pre-treated cells (Fig. 5e). Such findings emphasized on positive effects of ROS regulation on the plasticity of the rBM-MSCs.

**Fig. 6:**
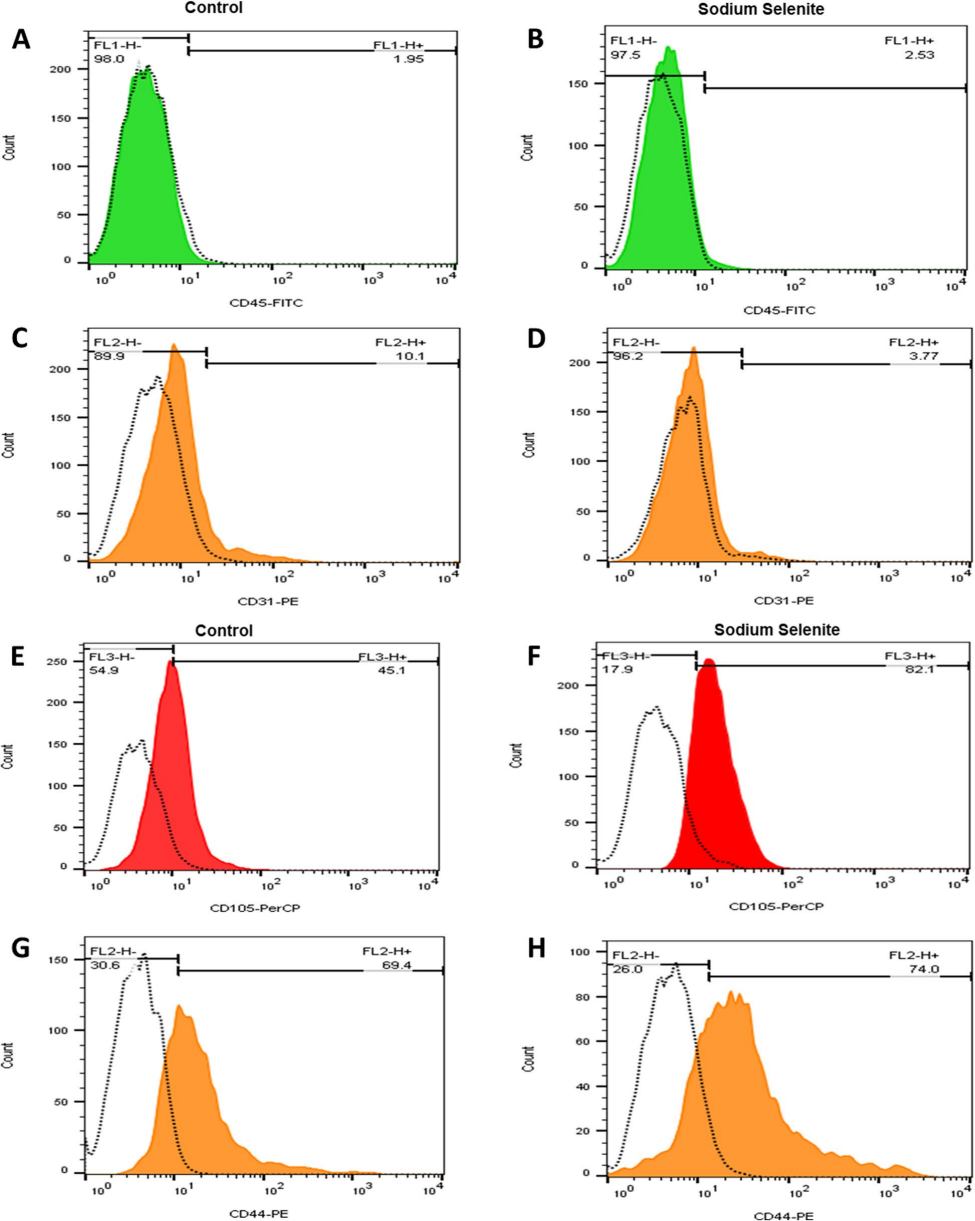
FACS characterization of markers after Sodium Selenite treatment of the rBM-MSCs. Based on the FACS analysis of the rBM-MSCs, the number of cells for CD44 and CD105 markers increased by about 4.6% and 37%, respectively, after sodium selenite treatment. FACS characterization of negative markers for control (**A,C, E,G**) and Sodium Selenite treatment (**B,D, F, H**) of the rBM-MSCs. Based on the FACS analysis of the rBM-MSCs, the number of cells for CD31 and CD45 markers was comparable. Number of samples = 3, Data: Mean ± SD

The cell surface markers expression of treated and untreated rBM-MSCs were reported in Fig. 6 and Table 2. According to the findings, most of the rBM-MSCs exhibited CD105 and CD44 (Fig. 6 E–H). Also, Sodium selenite did not induce any spontaneous differentiation-related changes in markers when used as a supplementation in routine culture (Table 2).

**Table 2:** Frequencies of rBM-MSCs stained for CD markers quantified by FACS

CD marker	CD31	CD45	CD44	CD105
Control	6.55 ± 0.35	9 ± 2.5	45.1 ± 2	68.9 ± 3.5
Sodium selenite	1.90 ± 0.45	4.01 ± 0.72	85.8 ± 5.3	75 ± 9

Number of samples = 3, Data: Mean ± SD

### Effect of sodium selenite on p38/MAPK/Nrf2 signaling pathway proteins

**Fig. 7:**
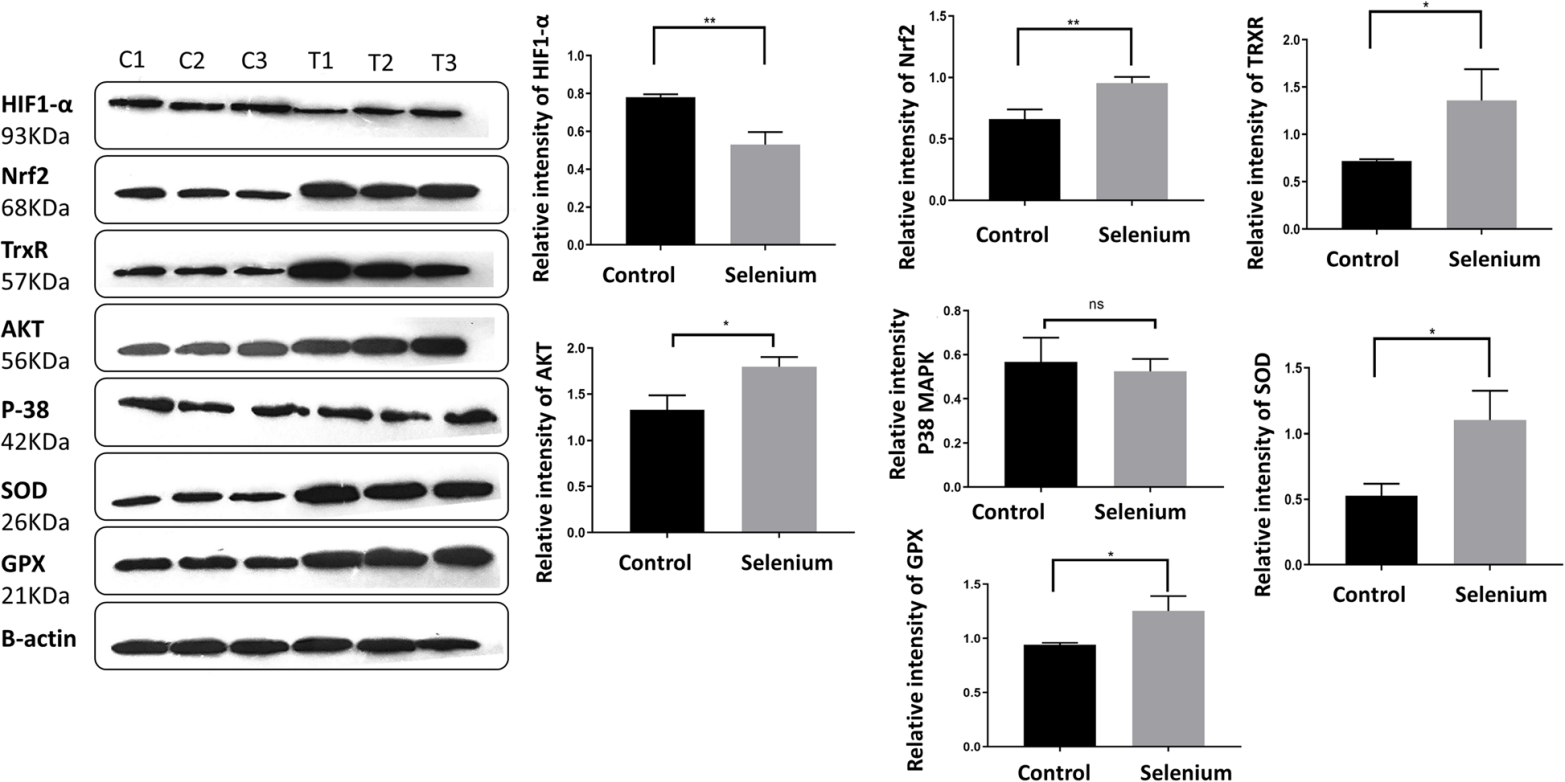
Western blot comparison of signaling molecules in response to Sodium Selenite (0.1 µM) in the rBM-MSCs. Expression of Nrf-2 and Selenium dependant antioxidant enzymes, such as SOD, GPX, and TrxR, increased compared with the control group. The level of AKT, P, increased after 72 h treatment with 0.1 µM sodium selenite. Data are represented as mean ± SD* *p* < 0.05, ** *p* < 0.01 significant level of treatment (T) vs. control (C) group. C1,2, and 3 are the three replicates of the control groups and T1,2, and 3 are three replicates of selenium (0.1 µM)treatment group. Full length blots are presented in the supplementary data. Number of samples = 3, Data: Mean ± SD

In the present study, the expression of the Nrf2 and its related enzymes was assessed by WB analysis. These proteins were selected by reviewing a bulk of relevant previously-published papers and considering our *in-silico* analysis results (3–7, 3–8 sections). Results of the WB analysis revealed a clear augmentation of the Nrf2 and other antioxidant enzymes, such as SOD, GPX, and TrxR, following sodium selenite exposure (*p*-Value < 0.05) (Fig. 7). To discover the effect of sodium selenite (0.1 µM) on ROS, the level of P38, the key protein in apoptosis activation, and the P38/MAPK signaling pathway and AKT (an essential protein in phosphorylation and deactivation of pro-apoptotic proteins) were also analyzed by WB. The results showed that after treatment with 0.1 µM sodium selenite, there were no significant differences between the control and treatment groups. The AKT level compared to the control group increased after 0.1 µM sodium selenite treatment(Fig. 7). The raw data for WB was included in the supplementary file (S1 and S2).

### Bioinformatics analysis of oxidative stress signaling pathways and Nrf2 Oxidative stress activates the MAPK/p38 and other signaling pathways

Based on our literature review of the previously-published papers in this research area, we provide a list of proteins and signaling pathways affected by Selenium supplementation. A comprehensive search strategy was applied, and because there were not many published reports, data were entered without any cell type or case limitation (Table 3).

**Table 3:** Pathways affected by selenium

Type of Cells	Signaling pathway	Effects of selenium	Ref
hMSCs	JNK/FOXO3 pathway	Increased JNK and FOXO3a expression	[62]
AF-MSCs	AKT-ERK1/2 Smad2 Stat3	Increased p-AKT,p-ERK,p-Smad2,and TGF-β expression	[1]
MSCs	ERK	Suppressed the activation of ERK induced by H2O2	[63]
osteosarcoma	p53/ATM/FOXO3a	Activation of p53	[64]
SSC	P21-mediated P53	-An increased abundance of P53, P21, P27 and BAX -Promote the proliferation by inhibiting ROS production	[65]
MSCs	BMP	Activation of the Smad-dependent BMP pathway, up-regulation of p-Smad1/5 protein, and down-regulation of PPARγ	[66]
3T3-L1	PI3K/Akt ERK	Activated the expression of the PI3K/Akt, as well as ERK	[67]
NPC	JNK/P38 MAPK, Akt	Blocks the activation of JNK/P38 MAPK, and Akt survival protein	[68]
CSCs	Nrf2 Wnt/β-catenin Notch	Suppression of β-catenin signaling	[69]
ATSCs	c-Jun Akt	Inhibition of reactive oxygen species-mediated phospho-stress-activated protein kinase/c-Jun N-terminal protein kinase activation-activation of Akt-downregulated p53 and p21	[70]
TM3	ROS/JNK /c-jun	Protective effects of Selenium against cadmium-induced apoptosis, through inhibition of both the ROS/JNK/ c-jun	[71]
Chicken’s kidney	PI3K/AKT/Bcl-2	protective effects of Selenium against cadmium-induced apoptosis, through activating the PI3K/AKT/Bcl-2	[72]
KBD patients	ERK	Stimulates the phosphorylation of the ERK signaling pathway	[73]
BALB/c murine macrophage cells	p38 MAPK NF-κB	Inhibiting the p38 MAPK pathway	[74]

Among the signaling pathways affected by Selenium, we focused on mitogen‐activated protein kinase (MAPK or MAP kinase) pathways (ERK, JNK, and p38 kinase) activated in response to oxidative stress through ROS production [62, 63]. ROS are thought to activate MAPK pathways based on their oxidation potentials. ROS may achieve this by oxidizing MAPK signaling proteins and inactivating MKPs [75]. ERK regulates ROS levels and activation of Nrf2, which upregulates the main antioxidant enzymes (SOD, TrxR, GPX). Furthermore, by activating JNK, ROS can also cause cellular survival by upregulating FoxO expression, which enhances the levels of antioxidant production, and SIRT1, leading to the inhibition of p53-dependent transcription [76, 77].

In oxidative conditions, Keap1 is attached to Nrf2 and prevents its translocation to the nucleus; this leads to subsequent overexpression of the downstream genes [78]. In response to inducing ROS, Nrf2 is activated by oxidation of the cysteine residues in Keap1 and subsequently Nrf2 dissociation. OS leads to disulfide bridges, the Nrf2/Keap1 complex structure alteration, and the Nrf2 detachment from keap1. Alternatively, it has been documented that ROS levels can be affected by increasing translation levels of antioxidant proteins for instance Cu–Zn-SOD, Mn-SOD, GPX, GST-pi, MT3, and FHC through the NF- κB pathway [79]. Moreover, Recent studies have shown that oxidants produced from mitochondria are important mediators of molecular signaling. The ROS released by the electron chain in mitochondria is implicated in the mitochondria-dependent apoptotic pathway that involves proapoptotic (Bax, BAK) and antiapoptotic (Bcl-2) protein binding (14–3-3), the release of cytochrome c, and p53 signaling activation, leading to cellular death [80]. Oxidative stress activates the MAPK signaling pathway. ERK, JNK, and p38 kinase activation in response to oxidative stress through ROS can have both prosurvival and proapoptotic effects. ROS-activated PLC-gamma and Src phosphorylate Ras and Raf, and ERK. Activated ERK activates Nrf2, which upregulates antioxidants. ROS also increase the expression of JNK and p38. Like ERK, JNK has both prosurvival and proapoptotic roles in response to oxidative stress. Activated JNK can promote cell survival via the activation of FoxO, which upregulates antioxidant production, and SIRT1, which inhibits p53-dependent transcription. Conversely, activated JNK can promote apoptosis via activities both in the cytoplasm and in the nucleus. In the cytoplasm, JNK negatively regulates antiapoptotic proteins, such as Bcl-xL [81].

**Fig. 8:**
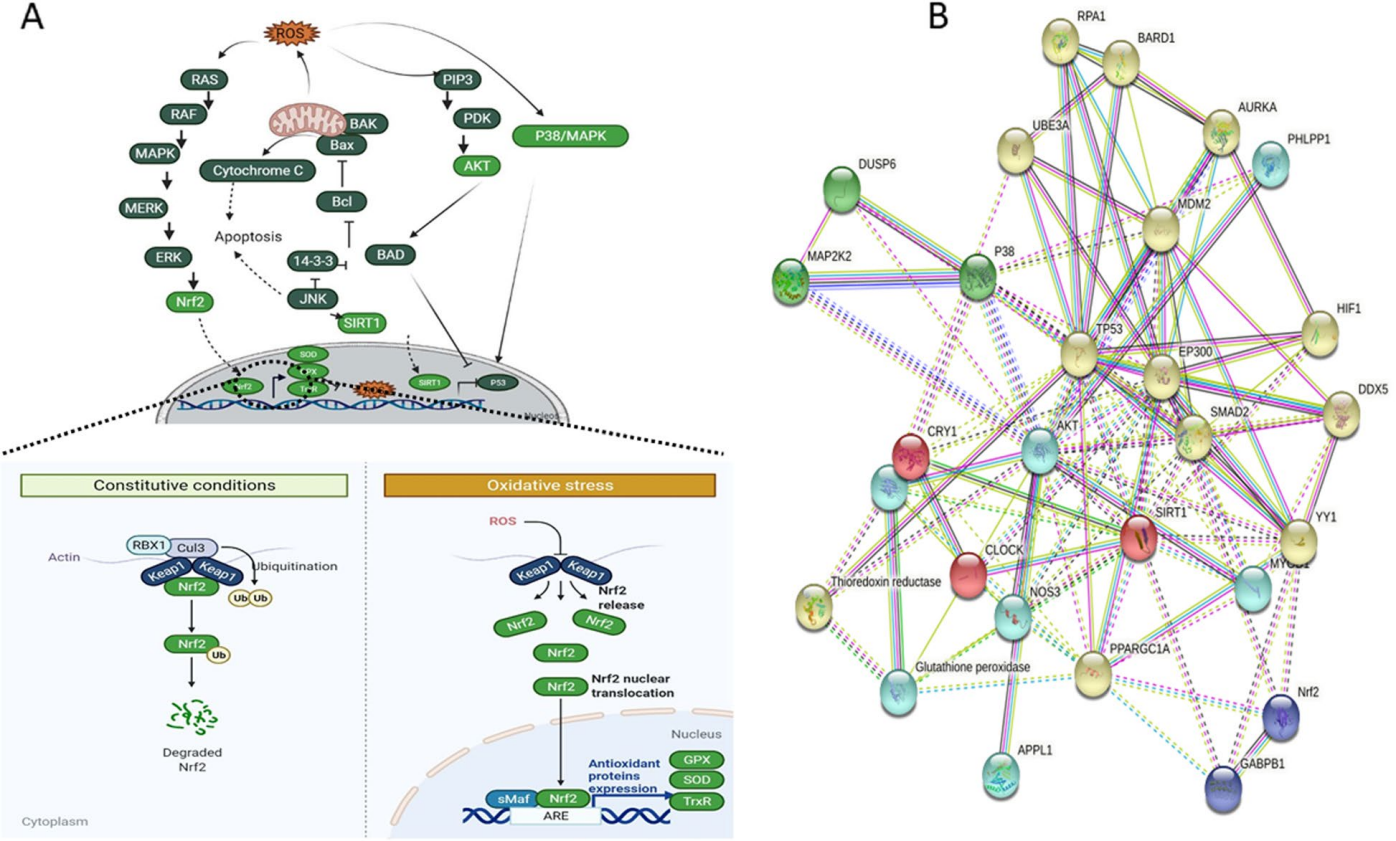
Schematic illustration of Nrf2 signaling pathway in oxidative stress (**A**) and protein network STRING (**B**). Nrf2-related signaling pathway plays a key role in directly regulating oxidative stress signaling pathway by overexpression of antioxidant enzymes (TrxR, SOD, GPX). Nrf2 is regulated by Keap1 in constitutive/oxidative conditions. Proteins coded light green were investigated in the present study, and those coded in dark green are bioinformatic predictions of the next layers in the network. The light green proteins was selected based theit ipmrtance in the siganling pathways, shown by the previously published reports (summerized in Table 3). The importance of these proteins were also confirmed by our by our bioinformatic analysis and pathway investigation by the String tool. **A** There are meaningful interactions among proteins (Nrf2, SOD, TrxR, GPX, SIRT1, AKT, P38, HIF1) based on the network and pathway analysis by STRING online tool. The different color lines show various interaction types among proteins on the system level. Dotted lines indicate inter-cluster and straight lines show cluster association (**B**)

**Fig. 9:**
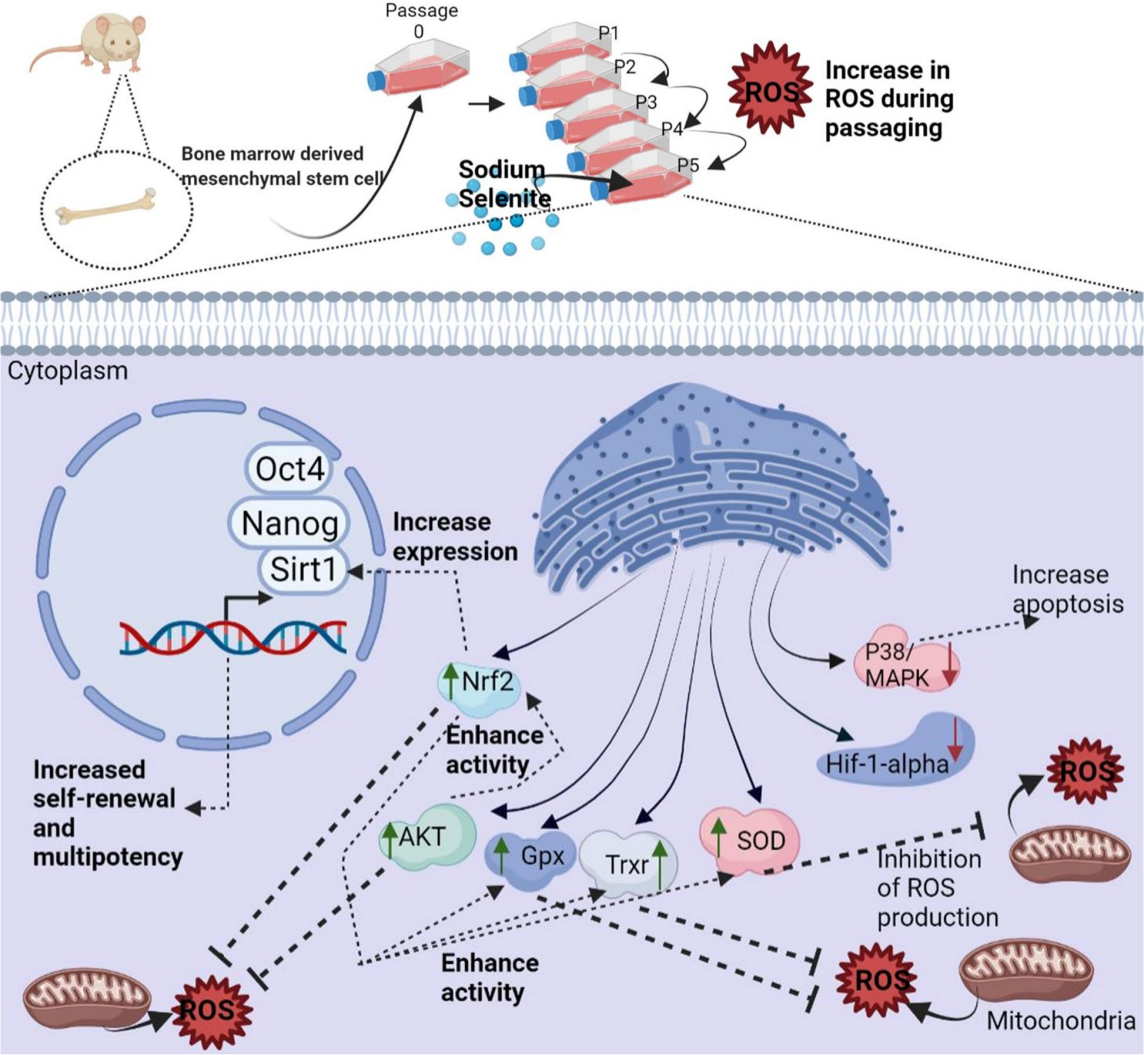
Supplementation of rBM-MSCs culture with sodium selenite caused Nrf2 overexpression, reduced the ROS level, improved cytoprotection by regulating the expression of HIF-1 of AKT, SOD, GPX, and TrxR markers and enhanced the expression of stemness related OCT-4, Sox2, Nanog. Number of samples = 3, Data: Mean ± SD

According to the DAVID database, the primary molecular function of these proteins is oxidoreductase. In the STRING database Nrf2, SOD, TrxR, GPX, AKT, P38, SIRT1, and P38 proteins were inserted in multiple proteins tab for homo sapiens and the signaling cascade has various topological characteristics, such as 28 nodes, 113 edges, 8.07 average node degrees, and 0.725 average local clustering coefficient. PPIs in this cascade have a confidence level of 0.400. The proteins are strongly connected with other proteins in the cascade. Therefore, in the present study, we investigated some of the most important proteins that are found in the Nrf2 signaling pathway and have an important role in oxidoreductase function (Nrf2, SOD, TrxR, GPX, AKT, P38, SIRT1, P38) found in defined signaling pathways and evaluated their role in Selenium treatment and ROS increment to determine the relationship between these pathways **(**Fig. 8**).** The number of the proteins involved in stress oxidative pathways were listed in Table 4. Overal results of this work were summarized in Fig. 9.

**Table 4:** The biological process involved in ROS stress regulation, according to STRING analysis

GO-term	Description	Count in network	Strength(> 1)
GO:0,090,400	Stress-induced premature senescence	2 of 8	2.43
GO:0,019,430	Removes superoxide radicals	2 of 13	2.22
GO:1,903,409	Biosynthesis of reactive oxygen species	3 of 25	2.12
GO:1,902,175	Regulating oxidative stress-induced intrinsic apoptotic signaling pathway	3 of 27	2.08
GO:1,902,176	Negative regulation of intrinsic apoptotic signaling pathways induced by oxidative stress	2 of 19	2.06
GO:0,098,869	Detoxification of cellular oxidants	4 of 90	1.68
GO:0,034,614	Cellular response to oxidative stress	5 of 133	1.61
GO:0,045,454	Cell redox homeostasis	2 of 60	1.56
GO:0,000,302	Response to reactive oxygen species	6 of 158	1.52
GO:2,000,379	positive regulation of the ROS metabolic process	3 of 103	1.5
GO:0,080,135	Regulatory mechanisms that control cellular responses to stress	9 of 739	1.12

## Discussion

In the basic research and clinical applications of stem cells, the cell sources must be plentiful and accessible and be efficiently delivered to the damaged sites. They also must be validated and optimized in the appropriate animal models, non-immunogenic and non-tumorigenic [82].

Among stem cell sources, MSCs have significant differentiation potential and could obtain easily from various tissues, including adipose tissue, bone marrow, and skeletal muscle [83]. Mainly, BM-MSCs are desirable cell sources due to their properties such as high proliferation, ease of access, harvesting simplicity, immune privilege, and differentiation potential into adipogenic, osteogenic, and chondrogenic lineages[84-86]. However, the stemness potential, differentiation capacity, and survival rate of cultured and transplanted stem cells to damaged sites could be limited by ROS-mediated oxidative stress imposed during isolation and in vitro expansion. This problem can lead to apoptosis and cell death in culture media and rejection of donor MSCs after transplantation [87, 88]. In addition, excessive ROS and oxidative stress could damage or modify cellular macromolecules, such as proteins, lipids, and DNA. Oxidative stress disrupts mitochondria, releases cytochrome C, and induces apoptosis by activating signal transduction pathways, such as P38 MAPK [12, 88]. The rat bone marrow-derived MSCs (rBM-MSCs) were employed for this work because they are available and user-friendly model cells in studies of basic stem cells and regenerative medicine [89]. The rBM-MSCs cells were isolated, cultured, and characterized and all results were in accordance with the International Society for Cell & Gene therapy (ISCT).

During the transplantation, the high mortality rate of BM-MSCs has been reported because oxidative stress-mediated apoptosis results in significantly decreased transplantation efficiency [90-92]. Mammalian cells have many anti-oxidative defense components to secure against oxidative stress. This incorporates the expression of specialized proteins such as catalase, superoxide dismutase, and a few peroxidases, as well as unspecific ROS-degrading antioxidants such as vitamin E, ascorbate, uric corrosive, carotenoids, or glutathione [93]. Therefore, using antioxidant supplements could increase the expression of exogenous antioxidant proteins and enzymes to improve the antioxidant ability of BM-MSCs, but their effect is limited [94]. Therefore, a valid strategy for protecting MSCs against in vitro acquired oxidative stress is necessary for entering these cells into clinical applications or getting real results in basic research. This study investigated sodium selenite as a ROS scavenging material to enhance the antioxidant ability of rBM-MSCs and reduce undesirable effects of oxidative stress [95]. Among methods coming to mind to protect MSCs, the cheapest and less challenging approach is the enrichment of media with available antioxidant elements. We selected Selenium since several reports of its positive impacts on MSCs were documented [66, 96]. However, few investigations performed on stemness, surface markers, differentiation potential, and probable signaling of Selenium mediated improvements of MSCs. In this work, the different doses of Sodium selenite were used to determine the optimum dose. The gene expression study using qRT-PCR showed that the transcription of NANOG and OCT-4 were significantly upregulated after Sodium selenite treatment.

Furthermore, supplementing rBM-MSCs culture with sodium selenite results in overexpression of Oct-4, NANOG, and SIRT1, which could improve the self-renewal and multipotency of rBM-MSCs. Previous studies have shown that regulation of p53 via Nrf2/p53/Sirt1 and OCT-4/Sirt1/p53 pathways leads to improved stemness ability of rBM-MSCs [97, 98]. Furthermore, p53 is a tumor suppressor and pro-apoptotic gene, which was recently identified to negatively mediates the stemness potential of stem cells [97, 99, 100].

SOX2, NANOG, and OCT-4 are stemness genes that maintain the multipotency and self-renewal of MSCs; it also is thought that Sirtuin 1 (Sirt1) has a role in the regulation of MSCs’ stemness ability. SIRT1 is a NAD-dependent protein deacetylase that controls several cellular mechanisms, including bone hemostasis, senescence, and metabolic pathways. In rBM-MSCs, Sirt1 can lead to long-term growth by reducing senescence during cell passages [97, 100]. Sirt1 activity can be suppressed by direct binding of P53 to its promoter, while Nrf2 can promote the degradation of p53 by regulating the expression of Mdm2, subsequently increasing the expression of Sirt1 and maintaining the stemness capacity of MSCs [97, 101].

Some studies indicated that Sirt1 regulates the expression of NANOG and OCT-4 by preventing p53 activation in human embryonic stem cells (hESC) and regulates SOX2 by post-translational modification in rBM-MSCs [98, 102]. In oxidative stress conditions, expression of Sirt1 prevents cytoplasmic translocation of p53 by its deacetylation, inhibits the p53-mediated repression of NANOG expression in stem cells, and maintains the stemness potential of stem cells [99, 100]. So increasing the level of Nrf2 protein after treatment with sodium selenite can maintain the undifferentiated state of rBM-MSCs via enhancing Sirt1 expression at the mRNA level. In addition, it was well known that OCT-4 preserves the stemness ability of stem cells by controlling NANOG and Sox2. OCT-4 maintains the stemness capacity of ESCs via inducing SIRT1, which inhibits p53 activity that induces ESCs differentiation through suppressing another stemness gene such as NANOG [98]. Aligned with these reports, our outcomes showed that sodium selenite could enhance the expression of stemness genes, including NANOG, OCT-4, and especially SIRT1 in mRNA level. The high level of SIRT1 is related to Nrf2- mediated p53 suppression and OCT-4-mediated inducing after treatment with sodium selenite in rBM-MSCs. Given the importance of these results in clinical applications, the role of sodium selenite in the maintenance of rBM-MSCs stemness potential is highlighted. On the one hand, overexpression of Nrf2 not only improves the antioxidant capacity [103] and survival rate of rBM-MSCs but could also regulate the stemness potential. Nevertheless, to date, no studies confirmed the association between Nrf2 and MSCs stemness potential or the interaction between Nrf2 and the expression of stemness genes [97]. Therefore, our study results were near to those of other mentioned studies and verified that sodium selenite could influence the rBM-MSCs in a positive manner.

Subsequently, the CD markers profile of rBM-MSCs were checked using flow cytometry. There are some reports regarding aged-associated lessening of CD markers profile [104, 105]. Moreover, some publications reported that MSCs surface markers decrease in higher passages [106] which subsequently, reduces the stemness and differentiation capacity and as a result, decreases the quality of MSCs. We selected 2 positive and 2 negative CD markers to check if Sodium selenite could preserve their decline during *in-vitro* culturing conditions. Interestingly, our results showed that Sodium selenite could improve CD markers expression profile of rBM-MSCs. Bouquest, et al. showed that CD31 negative MSCs up-regulate genes associated with stemness [107], thus we used this marker to check if could affect stemness potential. And, our results were in accordance to those of Bouquest. Also, Pham et al. investigated CD105 link with stemness and showed that the CD105 positive MSCs and CD105 negative MSCs have parallel stemness and differentiation potentials to three main lineages osteocyte, chondrocyte, and adipocytes [108, 109]. Previous report exhibited that CD44 expressed by MSCs has considered to play a role in stemness maintenance because of its involovment in contact between stem cells and progenitor cells within their niche [110].

Subsequently, the differentiation potential of rBM-MSCs was checked after Sodium selenite exposure, which exhibited enhanced adipogenesis in Sodium selenite pre-treated rBM-MSCs.

Our findings are in line with Wang et al., [111] who reported an increase in adipogenesis and fat deposition by Selenium pre-treatment. In line with our work previous reports documented that, sodium selenite improved chondrogenesis and osteogenesis of MSCs [56, 112].

The ROS levels of rBM-MSCs were investigated after Sodium selenite treatment. Sodium selenite supplementation reduced ROS levels in Cells.

In addition, previous studies confirmed that the overexpression of Nrf2 can improve MSCs’ resistance to oxidative stress and promote their survival. Nrf2 feels the level of intracellular ROS and can maintain the redox hemostasis under several conditions in MSCs. In response to oxidative stress, Nrf2 is activated and upregulates some downstream antioxidant and cytoprotective genes such as SOD and SIRT1 and some growth factors and cytokines like SDF-1 and VEGF, resulting in reduction or inhibition of apoptosis and senescence, and improvement of cell survival, migration, stemness maintenance, antioxidant capacity, and self-renewal of MSCs. [113]. Our results confirmed that supplementing sodium selenite into the culture medium could induce expression of Nrf2 protein in rBM-MSCs, followed by increasing the expression of SOD, GPX, and TrxR through Nrf2/ARE signaling pathway and ultimately decreased ROS level in rBM-MSCs.

Another investigated protein in cell survival and apoptosis is Akt. Akt, protein kinase B, is an essential component in the biological signaling pathway, such as the phosphoinositide 3-kinase (PI3K)/Akt signaling pathway, which prevents apoptosis, enhances cell survival, and improves angiogenesis and migration [114].

Kim et al. [115] reported that the AKT signaling pathway protected stem cells against oxidative stress damage by activating the Nrf2-ARE pathway. In addition, Martin et al. [116] indicated that Akt could increase the translocation of Nrf2 in the nucleus and bind to AREs by stabilizing of Nrf2 protein, which has a very short half-time. Also, they determined that activation of Akt increases stable protein levels, leading to enhanced Nrf2 protein levels. Besides, to improve the Nrf2 activity, Akt protein can decrease MSCs apoptosis and increase their survival rate through blocking the activity of pro-apoptotic protein Bax and enhancing anti-apoptotic protein Bcl-2.Based on our results, supplementation of sodium selenite could increase Akt protein level, improve antioxidant ability and stemness potential of rBM-MSCs by increasing Nrf2 stability and activity but also could decrease apoptosis rate of cells via regulation of Bax and BcL2.

Moreover, the MAPKs pathway was associated with apoptosis reduction of rBM-MSCs after treatment with sodium selenite. This pathway has critical functions in signal transduction and modulates stress responses and apoptosis, which their main subgroups include Jun N-terminal kinase (JNK), extracellular signal-regulated kinase (ERK), and p38 [117].

It was indicated that p38 is a common regulator of mitochondrial and ER stress-induced apoptosis through caspase-3 and caspase-12 in MSCs. Therefore, activation of p38 is contributed to early apoptosis due to oxidative stress [117]. We found that supplementing rBM-MSCs culture media with sodium selenite as an antioxidant doesn’t change the P38 related to the MAPK signaling pathway in a significant way in this study, the Hypoxia-inducible factor 1α (HIF1α) after treatment with sodium selenite was decreased, while previously indicated that HIF1α expression is increased in hypoxia condition, which is necessary for pluripotency and survival of stem cells in hypoxic condition [118-120]. Additionally, the role of HIF1α in hypoxia conditions be considered that could affect various cellular physiology processes such as anti-apoptosis, proliferation, and migration of MSCs [119]. As a result, based on our findings, HIF1α had not a vital role in oxidative stress.

Previous studies showed that HIF-1a transcription is amplified in oxidative stress [121] (in agreement with previous reports, we observed that HIF-1a protein levels were high in cells and were considerably reduced after Selenium treatment. Also, we observed that Nrf2 shows up-regulation and inversely down-regulation of HIF-1a. This data is compatible with a previous study, which documented opposite responses to oxidative stress [122].

## Conclusion

The final conclusion is that the ideal cytoprotection for rBM-MSCs during *in-vitro* experiments could be sodium selenite supplementation. Because, sodium selenite helps to maintain rBM-MSCs stemness capacity, differentiation potential, and expression of surface CD markers. Moreover, positively regulate antioxidant proteins expression via increased Nrf2 protein levels, leading to the suppression of oxidative stress. Our results verified the antioxidant and cytoprotective properties of sodium selenite on rBM-MSCs quality which may be mediated via the Nrf2 signaling pathway.

## Supplementary Information

**Additional file 1: S1. **The SDS-PAG gel before transfeering to membrane during western blot protocol.

**Additional file 2: S2. **The bands for western blot analysis after visualization by chemiluminscence.

## Data Availability

All data generated or analyzed during this study are included in this published article and its supplementary information files are available from the corresponding author on reasonable request.

## Abbreviations

BM-MSCs: Bone marrow-derived mesenchymal stem cells
MSCs: Mesenchymal stem cells
ROS: Reactive oxygen species
MTT: 3-(4,5-Dimethylthiazol-2-yl)-2,5-diphenyltetrazolium bromide
DCFH-DA: 2'-7'Dichlorofluorescin diacetate
FACS: Fluorescence-activated cell sorting
IF-1: Hypoxia-inducible factors
GPX: Glutathione peroxidase
SOD: Superoxide dismutase
TrxR: Thioredoxin reductase
p-AKT: Protein kinase B (PKB)
Nrf2: Nuclear factor erythroid 2-related factor 2
CD markers: Cluster of differentiation
OCT-4: Octamer-binding transcription factor 4
Sirt1: Sirtunin 1
Nrf2: Nuclear factor erythroid 2-related factor 2
OS: Oxidative stress
MAPK: Mitogen-activated protein kinase
PI3K: Phosphoinositide-3-kinase
PBS: Phosphate-buffered saline
PE: Phycoerythrin
FITC: Fluorescein isothiocyanate
FBS: Fetal bovine serum
DMSO: Dimethyl sulfoxide
RT-PCR: Reverse transcription polymerase chain reaction
DCFH-DA: 2′,7Dichloro-dihydro-fluorescein diacetate
DCFH: Dichloro-dihydro-fluorescein
DCF: 2',7'-Dichlorofluorescein
PI: Propidium iodide
DCF: Discounted cash flow
SDS-PAGE: Sodium dodecyl sulphate–polyacrylamide gel electrophoresis
PVDF: Polyvinylidene difluoride
BSA: Bovine serum albumin
TBST: Tris-buffered saline (TBS) plus Tween 20
HRP: Horseradish Peroxidase
ECL: Enhanced chemiluminescence
ERK: Extracellular signal-regulated kinas
JNK: Jun N-terminal kinase
ARE: Antioxidant response element
keap1: Kelch-like epichlorohydrin-associated protein 1
NAD: Nicotinamide adenine dinucleotide
Mdm2: Mouse double minute 2 homolog
hESC: Human embryonic stem cells
HIF1α: Hypoxia-inducible factor 1α
SSC: Spermatogonial stem cells
NPC: Neural progenitor cells
CSCs: Cancer stem cells
TM3: Mouse Leydig cell line
KBD: Kashin–Beck disease
