# Supplementary figures and images for: Sodium selenite preserves rBM-MSCs’ stemness, differentiation potential, and immunophenotype and protects them against oxidative stress via activation of the Nrf2 signaling pathway
Source: BMC Complement Med Ther. 2023 Apr 25;23:131. doi: 10.1186/s12906-023-03952-7 (PMC10127330; doi:10.1186/s12906-023-03952-7)

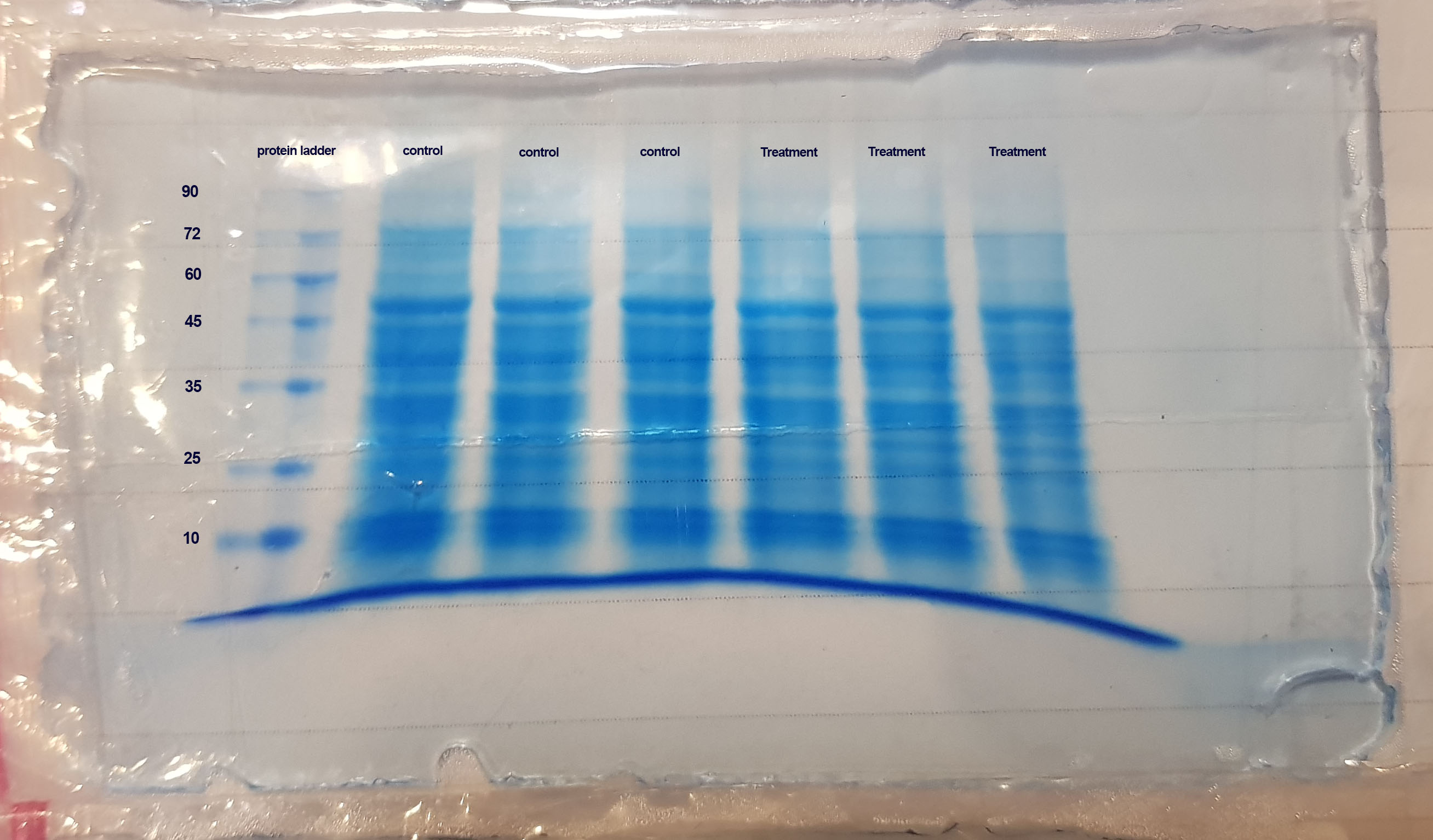

Supplement: Supplementary file 1 — Additional file 1: S1. The SDS-PAG gel before transfeering to membrane during western blot protocol. [file 12906_2023_3952_MOESM1_ESM.jpg]

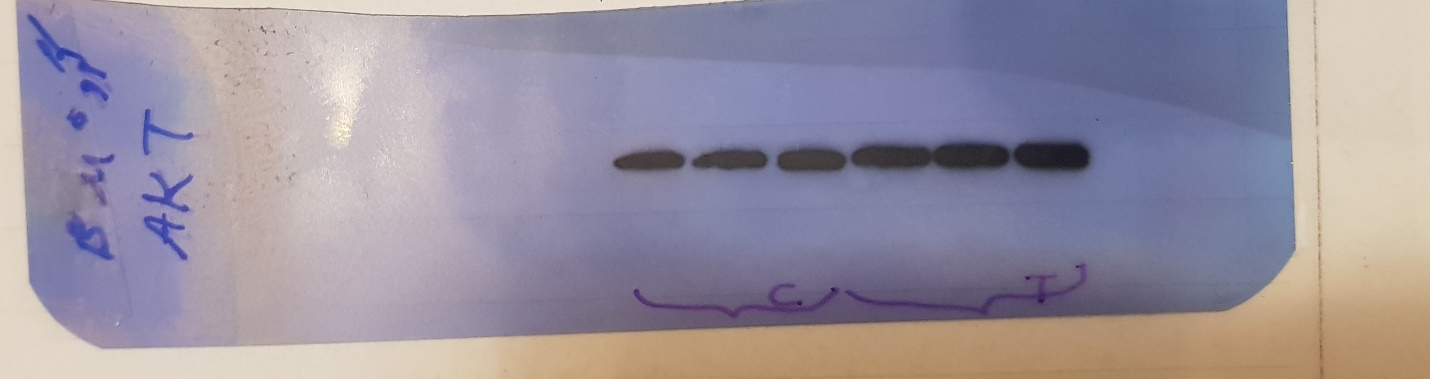


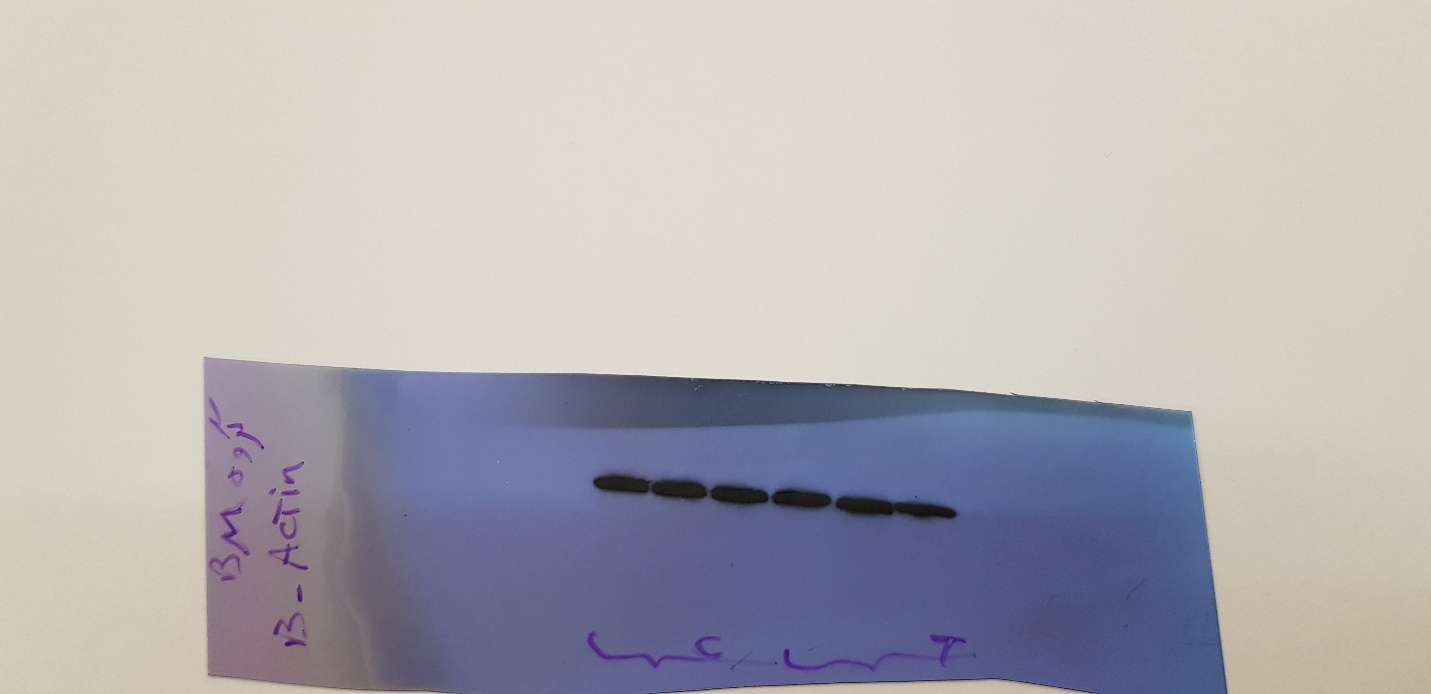


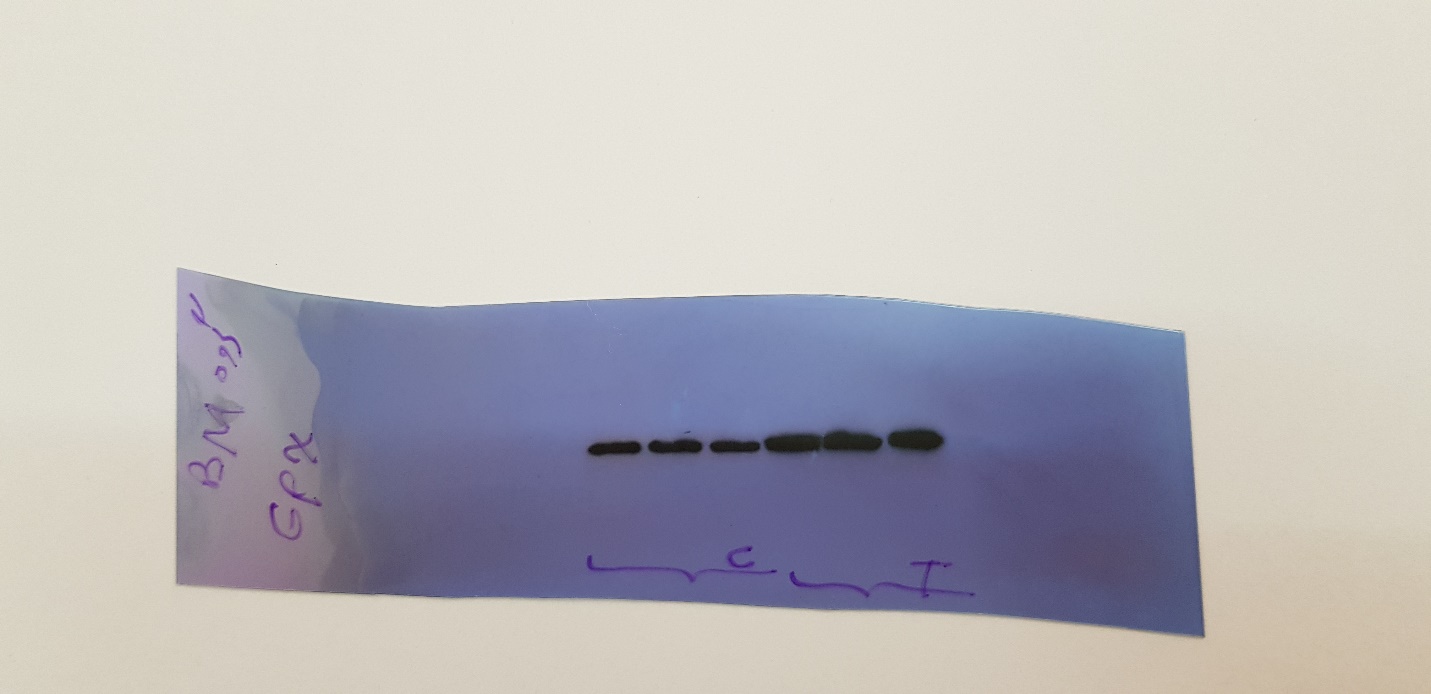


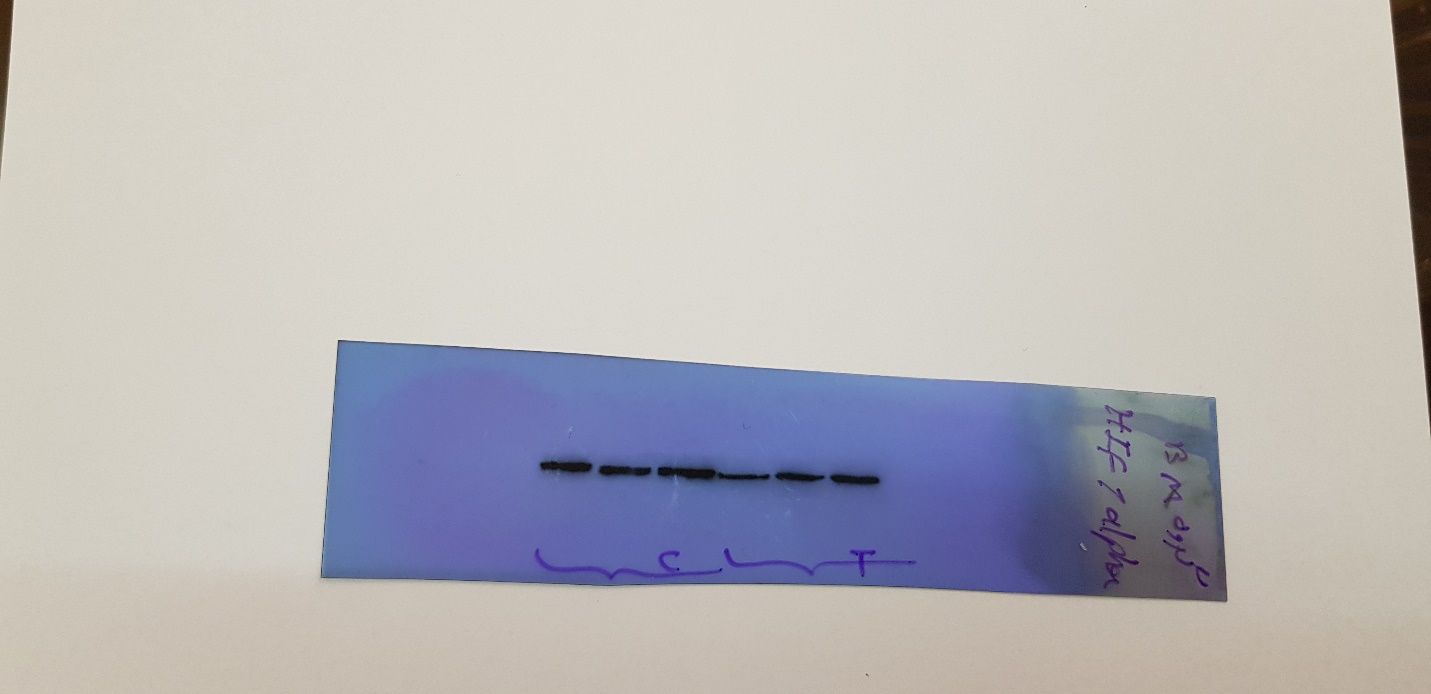


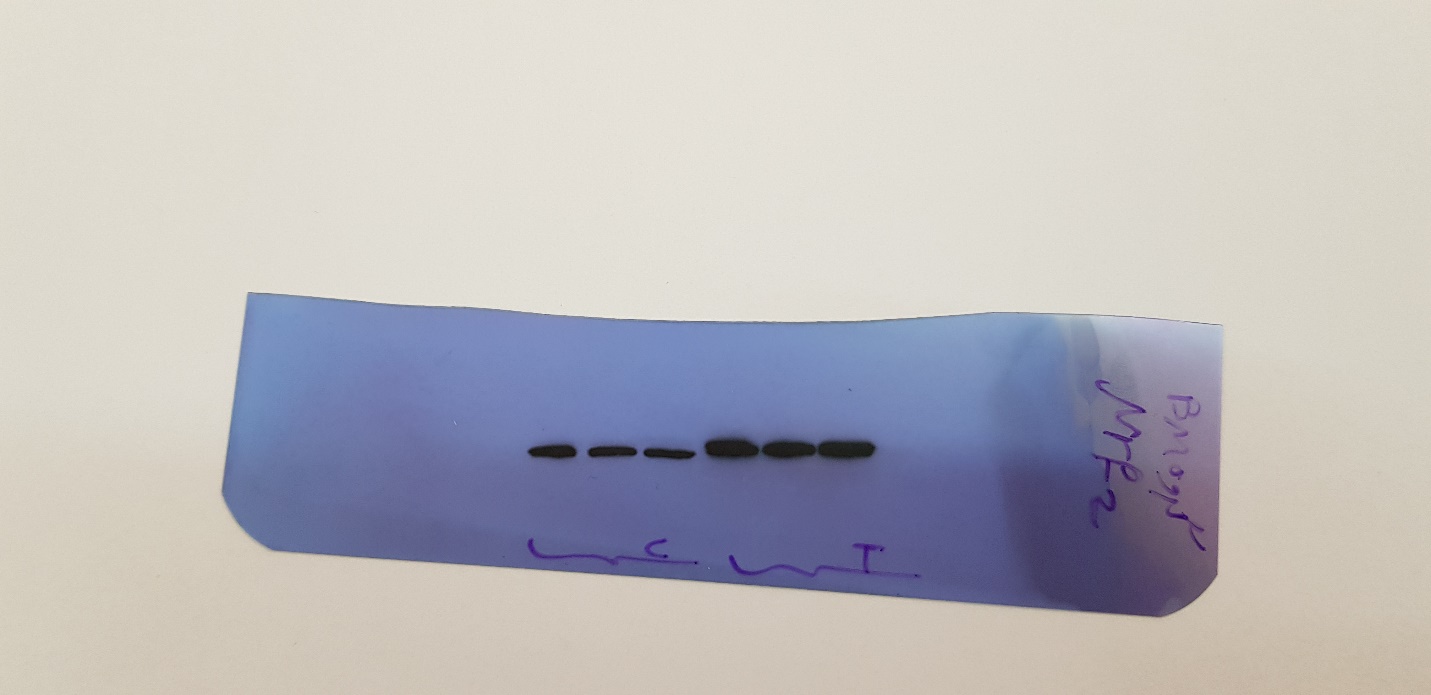


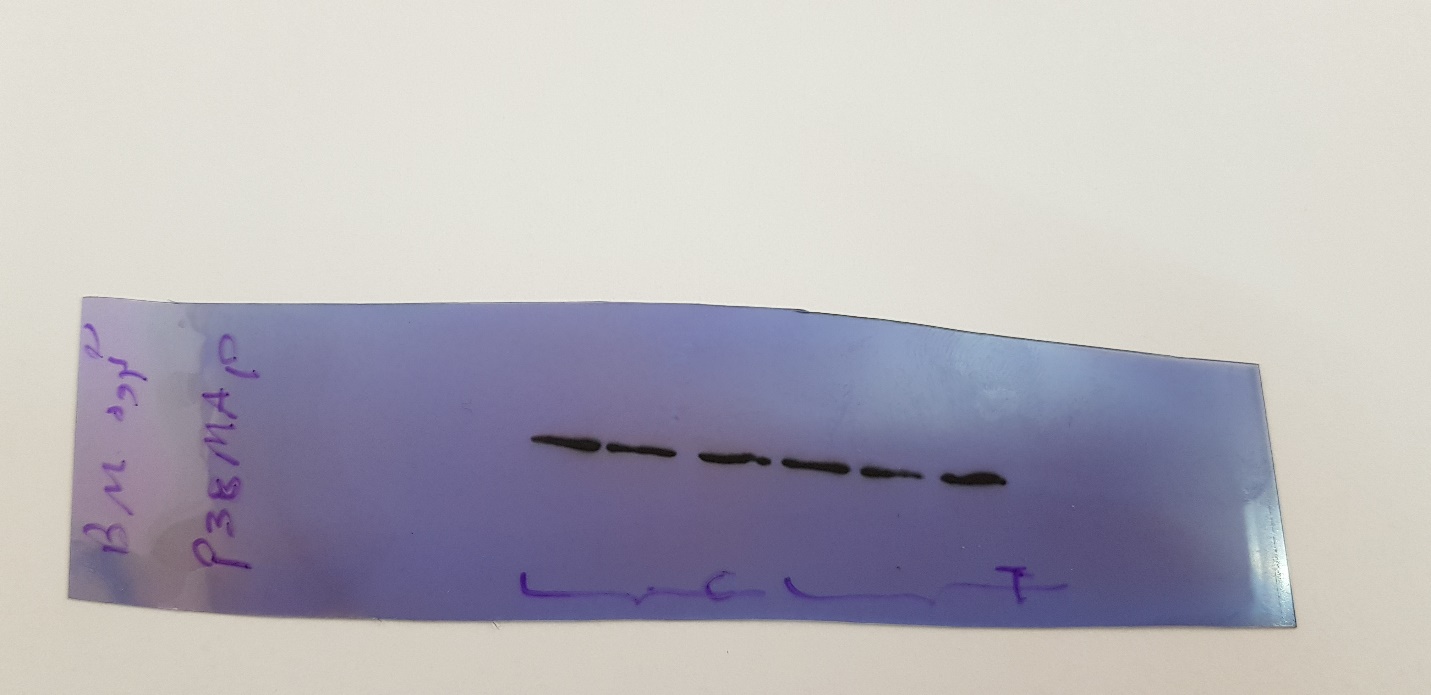


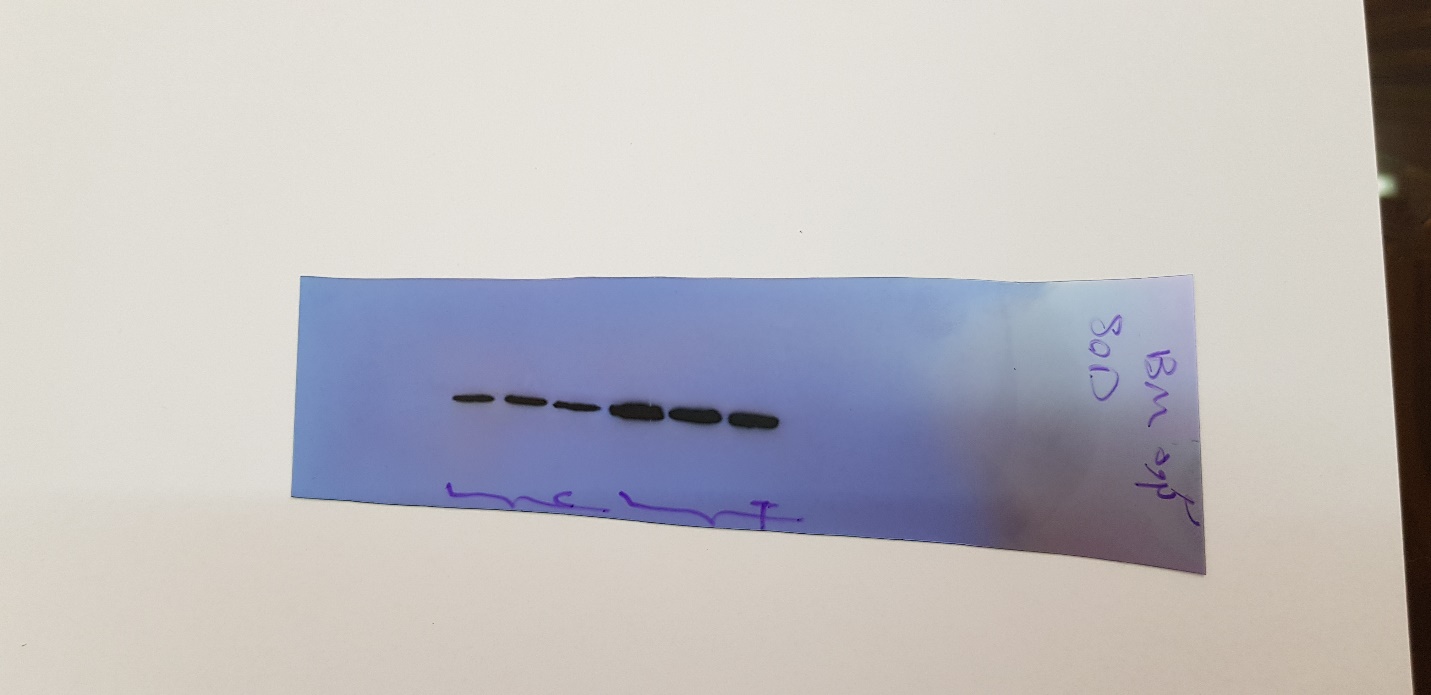


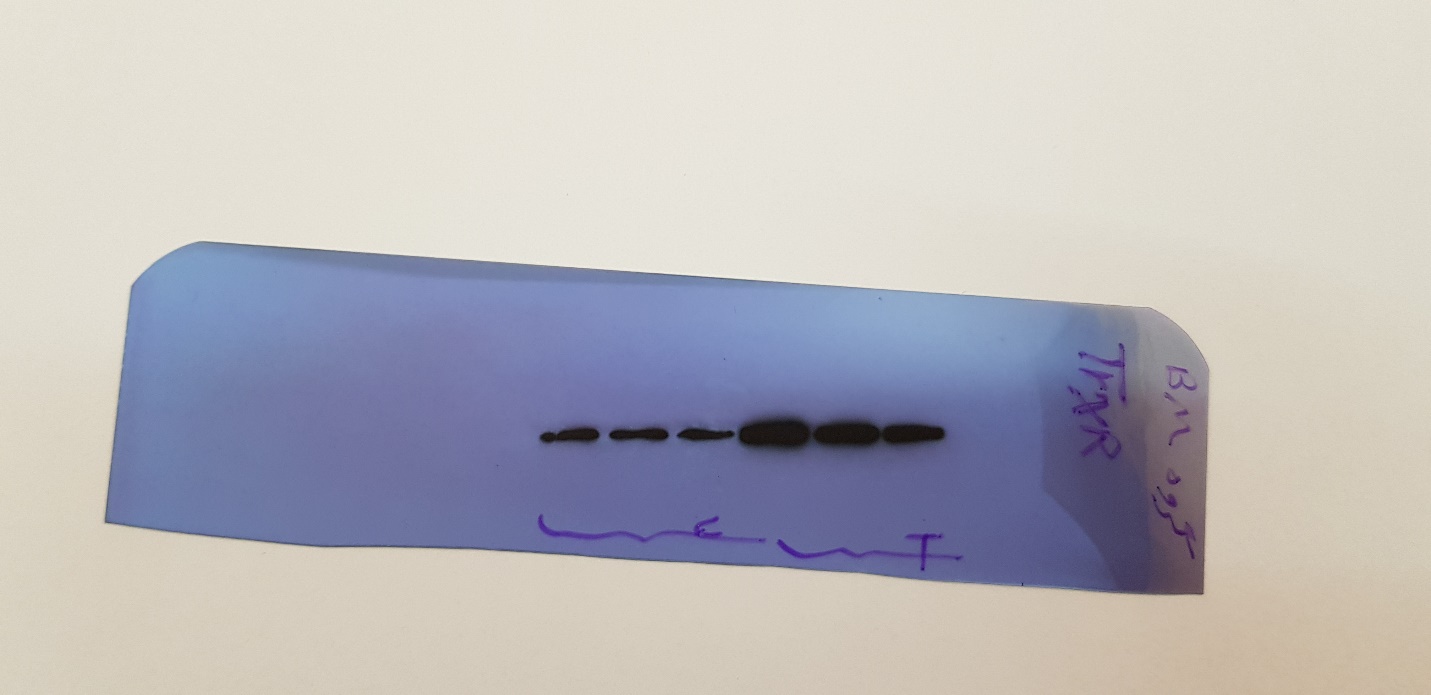


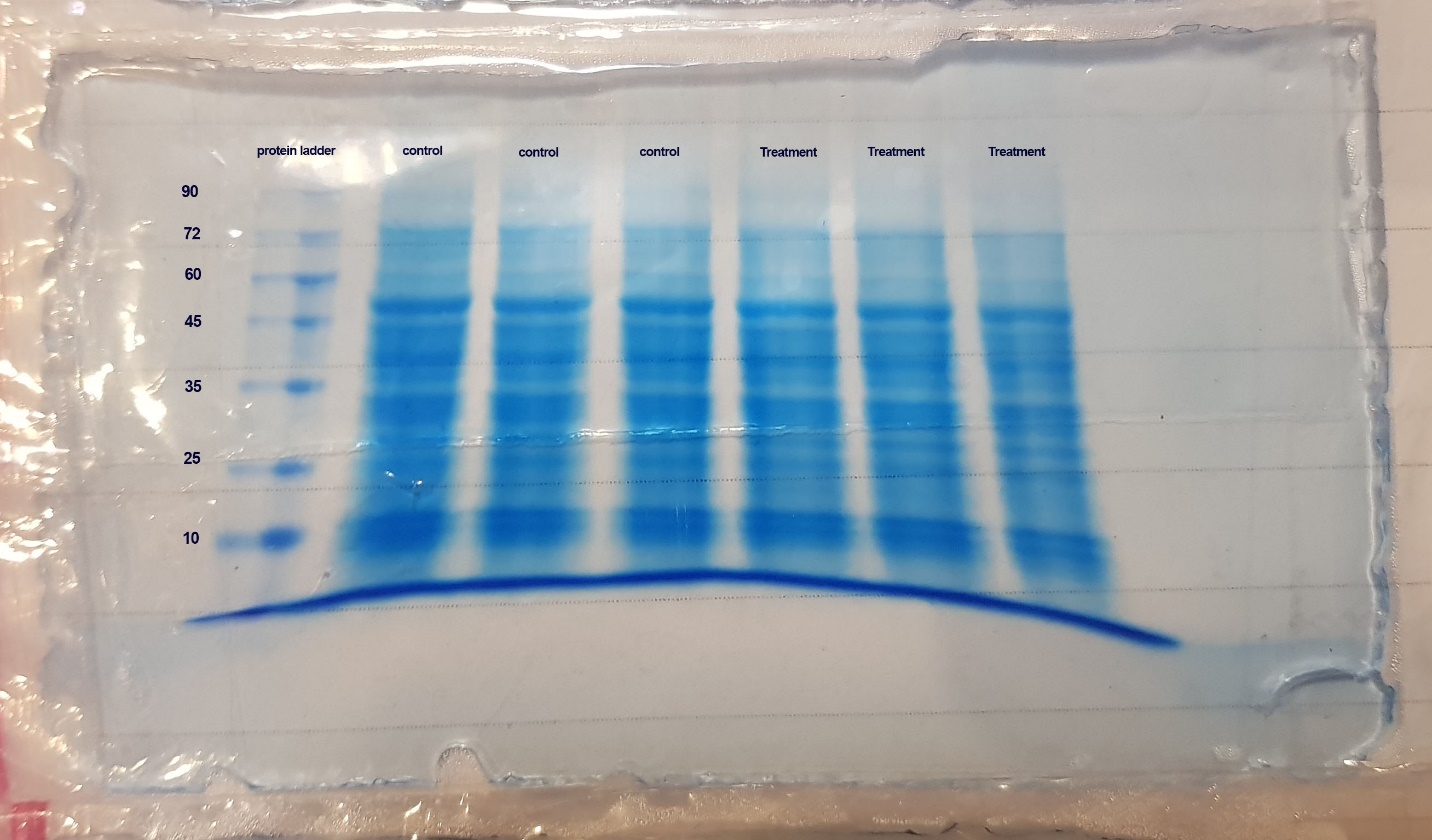

Supplement: Supplementary file 2 — Additional file 2: S2. The bands for western blot analysis after visualization by chemiluminscence. [file 12906_2023_3952_MOESM2_ESM.docx]
